# Pseudo-mono-axial ligand fields that support high energy barriers in triangular dodecahedral Dy(iii) single-ion magnets†

**DOI:** 10.1039/d2sc03182e

**Published:** 2022-10-31

**Authors:** Ben Zhang, Zhijie Cheng, Yingying Wu, Lei Chen, Rong Jing, Xingwei Cai, Chunhui Jiang, Yi-Quan Zhang, Aihua Yuan, Hui-Hui Cui, Zhao-Yang Li

**Affiliations:** School of Environmental and Chemical Engineering, Jiangsu University of Science and Technology Zhenjiang 212100 PR China chenlei@just.edu.cn aihua.yuan@just.edu.cn; School of Materials Science and Engineering, Nankai University 38 Tongyan Road, Haihe Educational Park Tianjin 300350 PR China zhaoyang@nankai.edu.cn; Jiangsu Key Laboratory for NSLSCS, School of Physical Science and Technology, Nanjing Normal University Nanjing 210023 PR China zhangyiquan@njnu.edu.cn; School of Chemistry and Chemical Engineering, Nantong University Jiangsu 226019 PR China

## Abstract

The synthesis of air-stable, high-performance single-molecule magnets (SMMs) is of great significance for their practical applications. Indeed, Ln complexes with high coordination numbers are satisfactorily air stable. However, such geometries easily produce spherical ligand fields that minimize magnetic anisotropy. Herein, we report the preparation of three air-stable eight-coordinate mononuclear Dy(iii) complexes with triangular dodecahedral geometries, namely, [Dy(BPA-TPA)Cl](BPh_4_)_2_ (1) and [Dy(BPA-TPA)(X)](BPh_4_)_2_·*n*CH_2_Cl_2_ (X = CH_3_O^−^ and *n* = 1 for 2; L = PhO^−^ and *n* = 2 for 3), using a novel design concept in which the bulky heptadentate [2,6-bis[bis(2-pyridylmethyl)amino]methyl]-pyridine (BPA-TPA) ligand enwraps the Dy(iii) ion through weak coordinate bonds leaving only a small vacancy for a negatively charged (Cl^−^), methoxy (CH_3_O^−^) or phenoxy (PhO^−^) moiety to occupy. Magnetic measurements reveal that the single-molecule magnet (SMM) property of complex 1 is actually poor, as there is almost no energy barrier. However, complexes 2 and 3 exhibit fascinating SMM behavior with high energy barriers (*U*_eff_ = 686 K for 2; 469 K for 3) and magnetic hysteresis temperatures up to 8 K, which is attributed to the pseudolinear ligand field generated by one strong, highly electrostatic Dy–O bond. *Ab initio* calculations were used to show the apparent difference in the magnetic dynamics of the three complexes, confirming that the pseudo-mono-axial ligand field has an important effect on high-performance SMMs compared with the local symmetry. This study not only presents the highest energy barrier for a triangular dodecahedral SMM but also highlights the enormous potential of the pseudolinear Dy–L ligand field for constructing promising SMMs.

## Abstract 2

Air-stable triangular dodecahedral Dy(iii) single-ion magnets with pseudo-mono-axial linear ligand fields exhibit high energy barrier exceeding 600 K, which represent the highest energy barrier for mononuclear SMMs with triangular dodecahedron.

## Graphical abstract

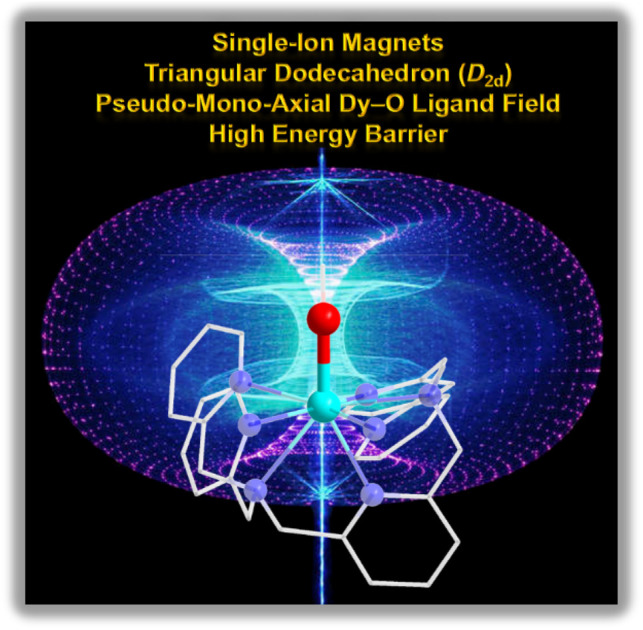

## Introduction

A single-molecule magnet (SMM) exhibits blocked magnetization below a critical temperature (*T*_B_) due to an anisotropy barrier (*U*_eff_). Such magnets have attracted unprecedented interest because they represent the ultimate size limit for future spin-based devices.^1–5^ Furthermore, obtaining SMMs with high *U*_eff_ and *T*_B_ values, ambient stabilities, and coercivities for technological applications is important.^6^ Mononuclear lanthanide single-molecule magnets (SMMs), also referred to as “single-ion magnets” (SIMs), are particularly preferred for realizing the goals of high-performance SMMs because the coordination environment of a single lanthanide ion can be manipulated to constantly refresh the records of *U*_eff_ and *T*_B_.^7–20^

A linear Ln(iii) complex (either one- or two-coordinated) is well known to provide an ideal coordination environment with a pure axial ligand field, which maximizes crystal field (CF) splitting and significantly improves axial doublets and magnetic properties.^21–26^ However, constructing such linear L–Ln–L or Ln–L complexes is synthetically challenging. Fortunately, this synthetically challenging geometry has been approached by constructing pseudolinear L–Ln–L complexes that possess two very short chemical bonds with an approximate angle of 180°, such as in octahedral (*O*_h_),^27–30^ pentagonal bipyramidal (*D*_5h_),^31–43^ hexagonal bipyramidal (*D*_6h_)^44–49^ and sandwich Dy(iii) complexes,^50–54^ resulting in impressive relaxation barriers and high blocking temperatures. Significantly, a dysprosium metallocene complex bearing cyclopentadienyl (Cp^R^) derivative ligands, as an example of the latter sandwich structure, has the most linear Cp^R^–Dy–Cp^R^ angle and short Dy–Cp^R^ distance, leading to a record hysteresis temperature of 80 K.^50^ Therefore, systems with pseudolinear L–Dy–L units have been shown to effectively produce high-performance SMMs. The other pseudolinear type of Dy–L complex, with only one very short coordinate bond, has rarely been explored, although computational studies suggest that such structures should possess significant energy barriers.^21^ Notable examples include [DyF(Tp^py^)(sol)*x*][PF_6_] (Tp^py^ = tris(3-(2-pyridyl)pyrazolyl)hydroborate; *x* = 1, 2) and [DyF(L^py^)](CF_3_SO_3_) (L^py^ = 1,4,7,10-tetrakis(2-pyridylmethyl)-1,4,7,10-tetraazacyclododecane), which are air-stable Dy–F complexes with two configurations, as reported by Norel and Canaj *et al.*^55–57^ Bulky polydentate ligands with six to eight donor atoms can chelate most of the Dy(iii) ion coordination sites in these complexes with weak coordinate bonds, leaving only small vacancies for fluoride to occupy.^58^ This system, with its dominant short dysprosium–fluoride bond, induces strong axial anisotropy and exhibits remarkable *U*_eff_ values (>600 K). In addition, these complexes generally exhibit high coordination numbers and are air stable as a consequence.

In this respect, our attention was drawn to the bulky heptadentate [2,6-bis[bis(2-pyridylmethyl)amino]methyl]pyridine (BPA-TPA) ligand^15^ that can enwrap metal centers, as demonstrated in the reported [Co(BPA-TPA)](A)_2_ (A = BF_4_, PF_6_ and BPh_4_) and [Co(BPA-TPA)](ClO_4_)_2_·H_2_O Co(ii) complexes.^59–63^ Monoanionic O-donor ligands, such as alkoxide, siloxide, and aryloxide, are distributed at axial sites and are particularly effective in high-performance Dy-based SMM complexes. Because most of the coordination sites of the Dy(iii) ion are occupied by a single bulky BPA-TPA ligand, we selected a sterically small methoxy (CH_3_O^−^) or phenoxy (PhO^−^) moiety as a candidate for forming a short, strong Dy–O bond. Herein, we report the synthesis, structural and magnetic characterization, and *ab initio* calculations of three novel air-stable complexes, [Dy(BPA-TPA)Cl](BPh_4_)_2_ (1) and [Dy(BPA-TPA)(X)](BPh_4_)_2_·*n*CH_2_Cl_2_ (X = CH_3_O^−^ and *n* = 1 for 2; L = PhO^−^ and *n* = 2 for 3), which possess a triangular dodecahedral coordinate geometry with *D*_2d_ symmetry. Dynamic magnetic susceptibility studies reveal that complex 1 only exhibits frequency-dependent out-of-phase (*χ*_M_′′) signals below 2.4 K under an 800 Oe dc field, whereas 2 and 3 are zero-field single-ion magnets with large energy barriers (*U*_eff_ = 686 K for 2; 469 K for 3); such magnets are unprecedented for the triangular dodecahedral geometry. Furthermore, complete-active-space self-consistent field (CASSCF) *ab initio* computational methods were used to provide detailed insight into the magnetic dynamics for these complexes and to understand the role of the axial Dy–O crystal field in realizing the observed excellent properties. This study provides a unique design approach toward a new class of complexes with the desired pseudolinear Dy–L ligand field.

## Results and discussion

### Crystal structures

**Fig. 1:**
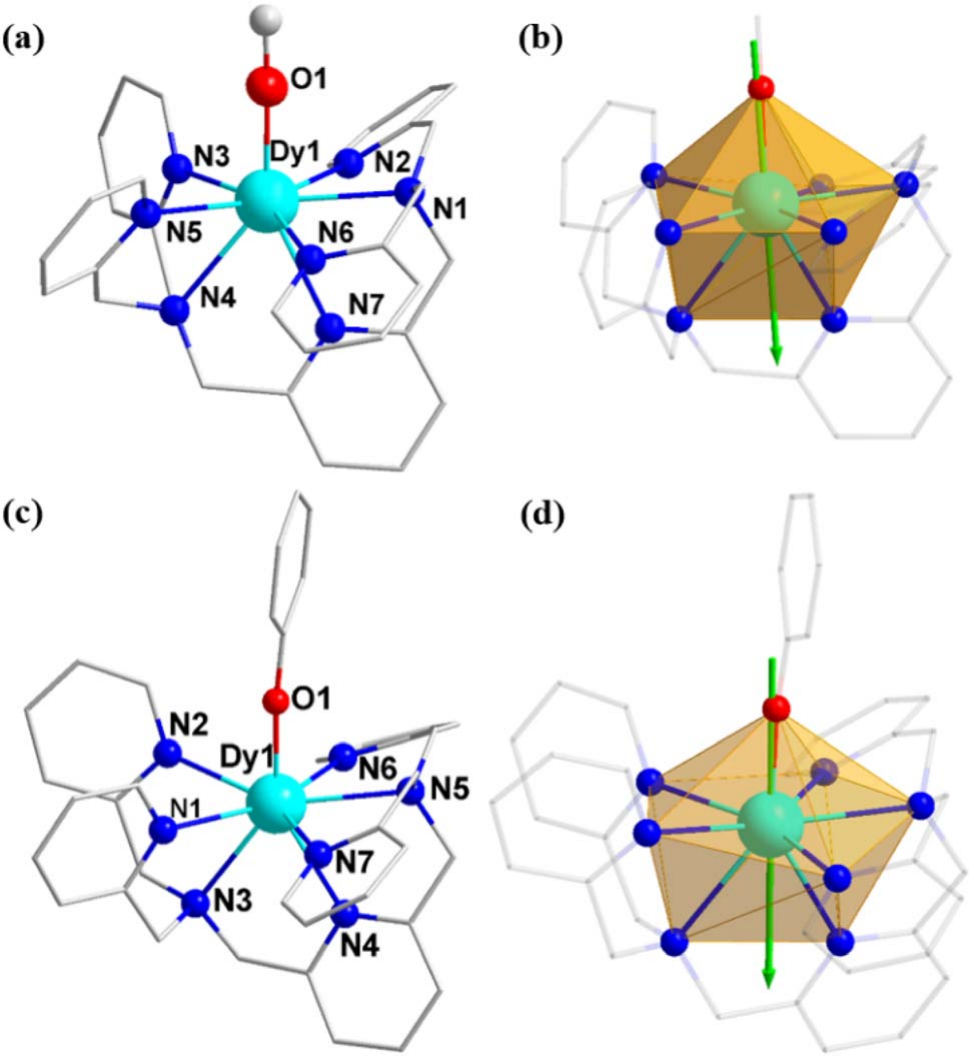
Coordinate structures and calculated orientations of the local main magnetic axes on Dy^III^ ions in the ground KDs of [Dy(BPA-TPA)(X)]^2+^ for complexes 2 (a and b) and 3 (c and d). Color scheme: Dy, cyan; N, blue; O, red; C, gray. H-atoms have been omitted for clarity.

The crystallographic structures of the three above mentioned complexes reveal that complex 1 crystallizes in the monoclinic *P*2_1_/*c* space group, while complexes 2 and 3 crystallize in the triclinic *P*1̄ space group (Table S1 in the ESI†). The coordinate structures of the cations of the three complexes are depicted in Fig. 1 and S4, with selected bond lengths given in Tables 1 and S2.†

**Table 1:** Selected bond lengths (Å) for 2 and 3

2		3	
**Dy(1)–O(1)**	**2.108(5)**	**Dy(1)–O(1)**	**2.087(3)**
Dy(1)–N(1)	2.523(5)	Dy(1)–N(1)	2.514(3)
Dy(1)–N(2)	2.616(6)	Dy(1)–N(2)	2.567(3)
Dy(1)–N(3)	2.495(5)	Dy(1)–N(3)	2.638(3)
Dy(1)–N(4)	2.616(5)	Dy(1)–N(4)	2.508(3)
Dy(1)–N(5)	2.555(5)	Dy(1)–N(5)	2.548(3)
Dy(1)–N(6)	2.602(5)	Dy(1)–N(6)	2.496(3)
Dy(1)–N(7)	2.507(5)	Dy(1)–N(7)	2.540(3)

The Dy(iii) site in complexes 1–3 is eight-coordinated by one BPA-TPA ligand and one Cl^−^, CH_3_O^−^ or PhO^−^ anion. The seven-nitrogen “pocket” of the BPA-TPA ligand three-dimensionally surrounds the central Dy(iii) ion with long Dy–N distances of 2.458(5)–2.619(5) Å for 1, 2.495(5)–2.616(6) Å for 2 and 2.496(3)–2.638(3) Å for 3, indicative of a fully weak crystal field in each case. The one remaining site is occupied by a CH_3_O^−^ or PhO^−^ anion with a very short Dy–O distance of 2.108(5) Å (for 2) or 2.087(3) Å (for 3), which induces an axially strong ligand field, whereas a long Dy–Cl axial bond of 2.5835(16) Å is observed for 1, suggesting a nearly spherical and weak ligand field. This allows us to unambiguously reveal that complexes 2 and 3 bear a pseudolinear Dy–O ligand field. Continuous shape measures (CShMs) were used to evaluate each configuration,^64–66^ the results of which are given in Table S3.† The triangular dodecahedral geometry (*D*_2d_) provided the lowest CShM value in each case: 2.166 for 1, 2.130 for 2 and 1.820 for 3. In addition, the shortest Dy(iii)⋯Dy(iii) distances were determined to be 10.770, 10.095 and 11.798 Å for 1–3, respectively, suggesting negligible direct and superexchange magnetic interactions (Fig. S8–S10†).

### Magnetic properties

**Fig. 2:**
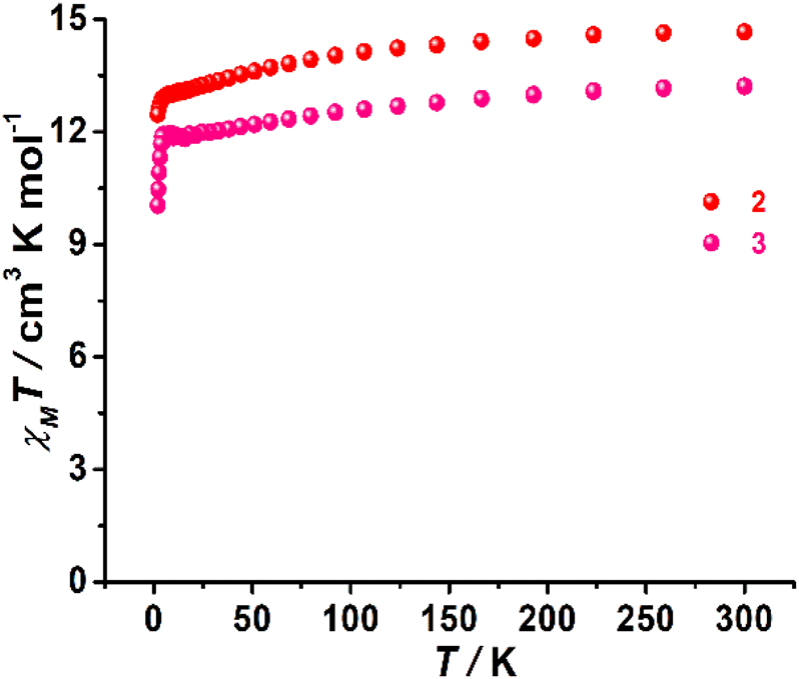
Variable-temperature dc susceptibility data for 2 and 3 in a 1000 Oe applied dc field.

Complexes 1–3 exhibited *χ*_M_*T* values of 13.65, 14.66 and 13.21 cm^3^ K mol^−1^, respectively, at room temperature, which are close to the theoretical value of 14.17 cm^3^ K mol^−1^ for an isolated Dy(iii) ion (*g* = 4/3, ^6^H_15/2_, *S* = 5/2, *L* = 5). These *χ*_M_*T* values decreased slightly upon cooling and then suddenly dropped at low temperature to final values of 6.98 cm^3^ K mol^−1^ for 1, 12.47 cm^3^ K mol^−1^ for 2, and 10.04 cm^3^ K mol^−1^ for 3 (Fig. 2 and S11†), which were likely attributed to thermal depopulation of *M*_J_ sublevels or the presence of large crystal field (CF) splitting. Field-dependent magnetization (*M*) curves were also acquired for three complexes at 2 K in the 0–7 T dc field range, which revealed respective magnetizations of 6.27 Nβ for 1, 6.11 Nβ for 2 and 5.06 Nβ for 3 at 7 T and 2.0 K (Fig. S12–S14†).

Dynamic magnetic relaxation was investigated by measuring the alternating current (ac) magnetic susceptibilities of 1–3 in the 1–999 Hz range under a zero external dc field. In the case of 1, no maximum of out-of-phase (*χ*_M_′′) susceptibilities was observed in the absence of a dc field (Fig. S15†). Further ac measurements were carried out under various dc fields. When applying the dc field, the *χ*_M_′′ signals only exhibit frequency dependence without the maximum. The temperature and frequency dependence of the ac susceptibilities under an 800 Oe dc field show the poor performance that is not *χ*_M_′′ signals above 2.4 K for 1 (Fig. S16†).

**Fig. 3:**
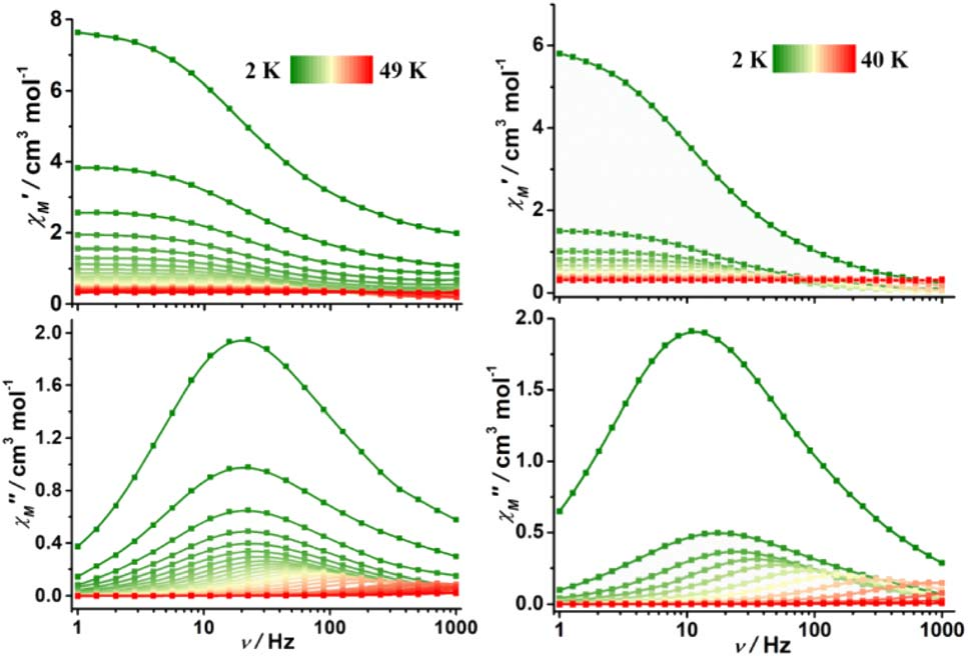
Frequency dependence of in-phase (top) and out-of-phase (bottom) ac susceptibility for 2 (left) and 3 (right) in zero dc field; the solid lines are guides for the eye.

**Fig. 4:**
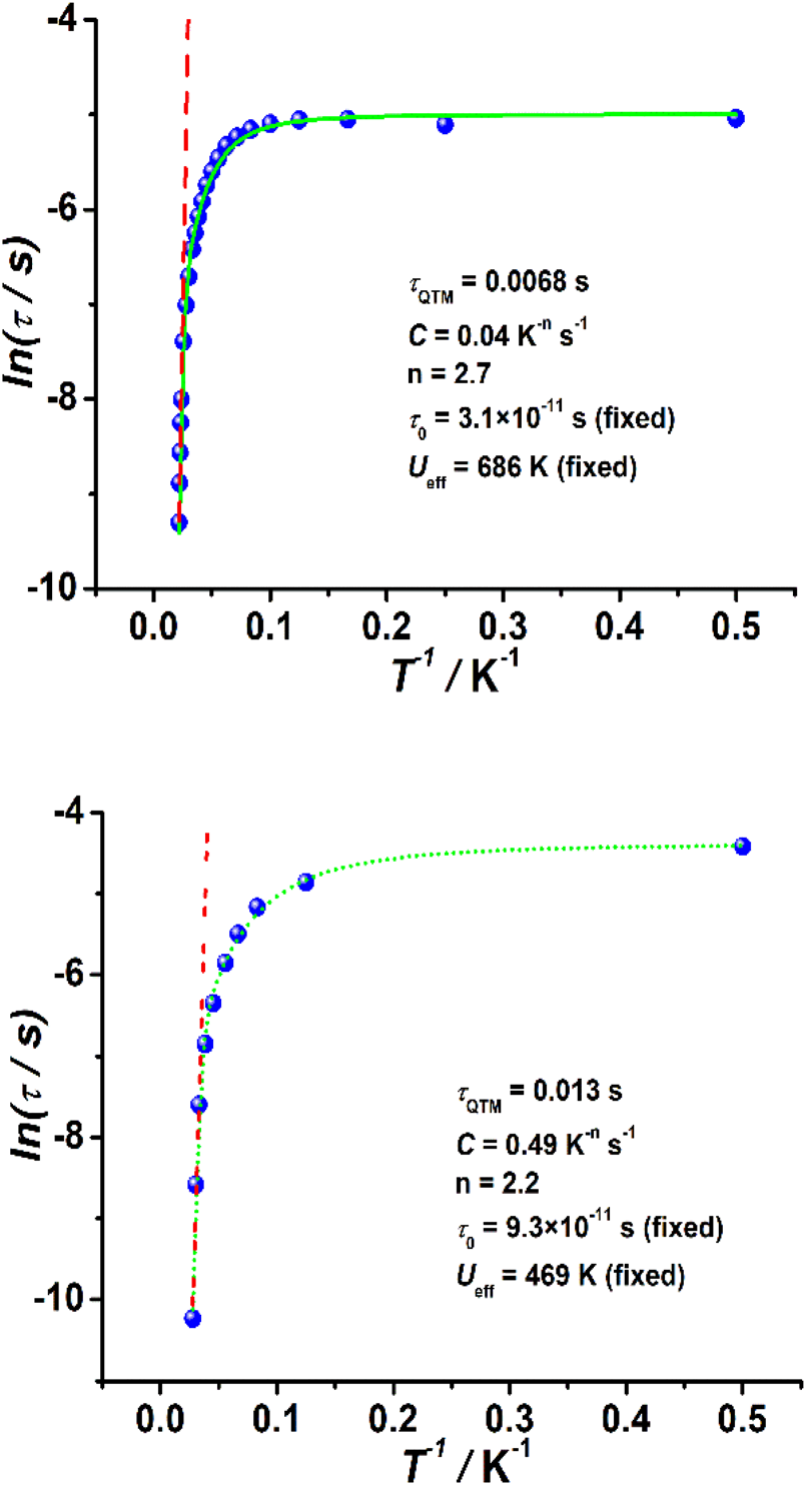
Plots of ln(*τ*) as functions of *T*^−1^ in a zero dc field for 2 (top) and 3 (bottom). The green solid lines are best fits to eqn (1), while the red dotted lines correspond to the Arrhenius law at high temperatures.

In the case of complexes 2 and 3, the in-phase (*χ*_M_′) and out-of-phase (*χ*_M_′′) ac susceptibility components show typical SMM behavior under zero dc field (Fig. 3, S17 and S18†). Well-defined *χ*_M_′′ maxima were observed for 2 and 3 at temperatures above 45 and 34 K, respectively, indicative of high barriers for magnetization reversal. Invariable *χ*_M_′′ (*ν*) peak maxima and *χ*_M_′′ (*T*) “tails” were observed below 10 K, consistent with the quantum tunneling of magnetization (QTM) that is commonly observed in lanthanide-based SMMs.^31–43^ The relaxation time (*τ*) extracted using the generalized Debye model^67–69^ obeys the Arrhenius law at high temperatures (Fig. S19 and S20, Tables S4 and S5†); linear regression provided the following best-fitted results: *U*_eff_ = 686 K and *τ*_0_ = 3.1 × 10^−11^ s for 2 and *U*_eff_ = 469 K and *τ*_0_ = 9.3 × 10^−11^ s for 3 (Fig. 4). To the best of our knowledge, these are the highest energy barriers for SIMs with triangular dodecahedrons and highlight the potential of the unique pseudolinear Dy–L ligand field.

Prominent QTM was observed below 10 K in each case. Indeed, Raman processes make significant contributions in most reported SIMs. In addition, direct processes can be neglected for relaxation time products in a zero dc field. Therefore, the magnetization dynamics of 2 and 3 can be fitted using eqn (1), which considers the QTM, Raman, and Orbach mechanisms:

1 *τ*^−1^ = *τ*_QTM_^−1^ + *CT*^*n*^ + *τ*_0_^−1^ exp(−*U*_eff_/*k*_B_*T*)

To avoid overparameterization, the fixed values of *U*_eff_ and *τ*_0_ obtained from the Arrhenius law were applied in the fit processes, yielding *τ*_QTM_ = 0.0068 s, *C* = 0.04 s^−1^ K^−*n*^, *n* = 2.7, *U*_eff_ = 686 K (fixed) and *τ*_0_ = 3.1 × 10^−11^ s (fixed) for 2, and *τ*_QTM_ = 0.013 s, *C* = 0.49 s^−1^ K^−*n*^, *n* = 2.2, *U*_eff_ = 469 K (fixed) and *τ*_0_ = 9.3 × 10^−11^ s (fixed) for 3, as shown in Fig. 4. The values of the Raman parameters *C* and *n* are within the ranges observed for Dy-based SMMs.

Upon applying the optimized field of 1500 Oe, the ac susceptibility data for both complexes show temperature dependence in the whole temperature region (Fig. S21–S24†) due to the suppression of the QTM at low temperature. The *χ*_M_′′ peak is located at 45 K for 2 and 34 K for 3, revealing almost unchanged relaxation at high temperature with respect to the zero-field data. These frequency-dependent data were also analyzed by the generalized Debye model (Fig. S25–S26 and Tables S6 and S7†). The Arrhenius fit at high temperatures generates *U*_eff_ = 688 K and *τ*_0_ = 3.8 × 10^−11^ s for 2 and *U*_eff_ = 492 K and *τ*_0_ = 6.7 × 10^−11^ s for 3 (Fig. S27 and S28†). These values of the Orbach parameters were employed for the fit based on the combination of the Raman and Orbach processes, as shown in Fig. S29 and S30.† The energy barrier values for 2 and 3 under 1500 Oe are in keeping with those for zero dc field, suggesting the Raman and Orbach processes at the high temperature region are unacted on the application of dc field.

**Fig. 5:**
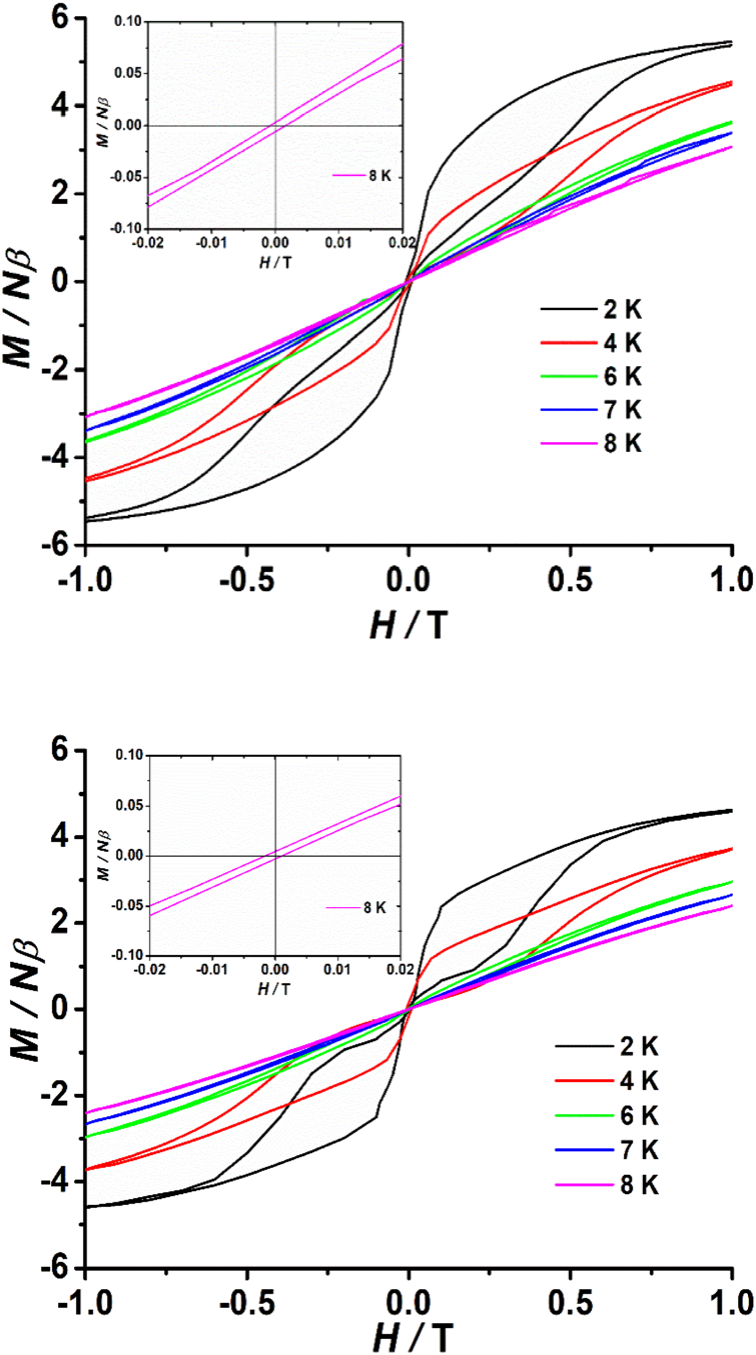
Powder magnetic hysteresis data for 2 (top) and 3 (bottom) at an average sweep rate of 0.02 T s^−1^. Inset: *M*(*H*) loops in the zero field region open up to 8 K.

To investigate the blocking of magnetization, polycrystalline samples of complexes 1–3 were subjected to magnetic hysteresis at a slow average sweep rate of 200 Oe s^−1^. It can be expected that no hysteresis loop was found at 2 K for complex 1 (Fig. S31†). In contrast, clear butterfly shaped hysteresis loops that open below 8 K were observed for complexes 2 and 3 (Fig. 5). Magnetization is observed to suddenly drop at low fields that approach *H* = 0, which reveals the strong contribution from a faster QTM effect, in good agreement with the temperature independence observed below 10 K in the *χ*_M_′′ (*ν*) curves and the rapid increase in *χ*_M_′′ (*T*) at low temperatures observed for 2 and 3.

### Theoretical analysis

To gain further insight into the magnetic anisotropies and relaxation mechanisms of complexes 1–3, CASSCF calculations on the basis of their X-ray determined geometries were carried out with the OpenMolcas and SINGLE_ANISO programs.^70–73^ For complex 1, a very small energy gap between the ground state and first excited states of 18.5 cm^−1^ was predicted. Relatively large *g*_*x*,*y*_ values of 0.428 and 3.426 with the impure ground state of *m*_J_ = 63%|±15/2 were observed, yielding a large magnetic moment matrix element of 0.64 *μ*_B_ between the ground Kramers doublets (KDs), which indicates that probable magnetic relaxation occurs *via* the ground state. These computed results explain the weak ac magnetic property even under the applied dc field for 1.

**Fig. 6:**
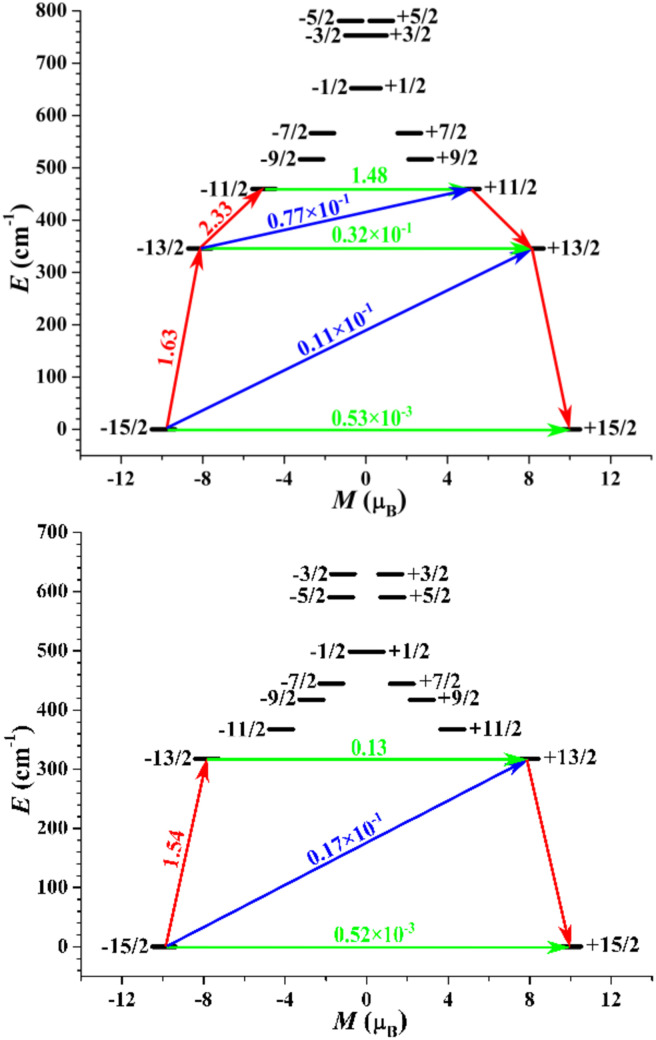
Magnetization-blocking barriers for complexes 2 (top) and 3 (bottom). The thick black lines represent KDs as a function of magnetic moment along their magnetic axes. The green lines correspond to diagonal QTM, while the blue lines represent off-diagonal relaxation processes. The paths shown by the red arrows represent the most likely paths for magnetic relaxation in the corresponding compounds. The number associated with each arrow is the mean absolute value of the corresponding matrix element of the transition magnetic moment.

In contrast to complex 1, a highly anisotropic ground Kramers doublet (KD) with *g*_*z*_ ≈ 19.86 was obtained for complexes 2 and 3 together with the first excited KD assigned to a rather pure *m*_J_ = ±13/2 state, whereas other KDs show substantial magnetic state mixing (Tables S5 and S6†). The main magnetic axes on the Dy^III^ ions of 2 and 3 in their ground KDs are shown in Fig. 1b and d; each lies exactly along the Dy–O direction and reveals the strong crystal field resulting from the short, strong Dy–O bond. The magnetization-blocking barriers of complexes 2 and 3 are shown in Fig. 6, where both ground KD transversal magnetic moments are smaller than 10^−3^*μ*_B_; hence, the QTM in their ground KDs is suppressed at low temperature. Complex 2 exhibits a transversal magnetic moment in the first excited KD of 0.32 × 10^−1^*μ*_B_; therefore, relaxation probably proceeds through the second excited KD. On the other hand, complex 3 shows a transversal magnetic moment in the first excited KD of 0.13 *μ*_B_; consequently, fast QTM is likely to occur in its first excited KD. The transversal magnetic moment in the second excited KD of 2 was found to be 1.48 *μ*_B_; therefore, fast QTM is expected to occur in its second excited KD. Accordingly, the energy barriers for 2 and 3 calculated according to the schemes in Fig. 6 are 459.6 and 317.5 cm^−1^, respectively, which agree well with the experimental values of 476.8 cm^−1^ (686 K) and 326 cm^−1^ (469 K).

**Fig. 7:**
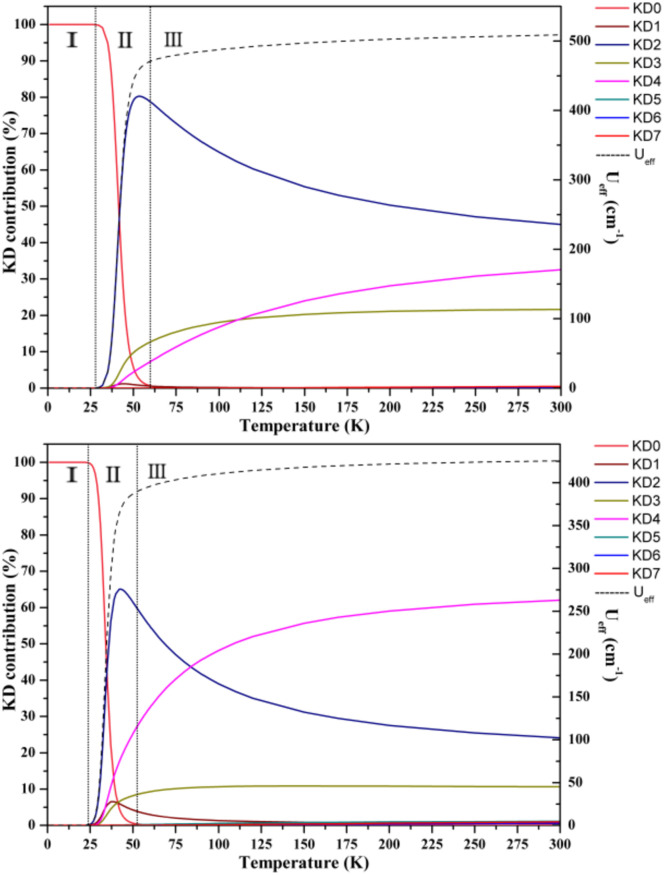
Predicted effective barrier and relaxation contributions from various KDs of complexes 2 (left) and 3 (right). Each *U*_eff_ is represented as a dashed black line, and its values are indicated on the right *y*-axis. The left *y*-axis represents the relative contribution of each KD to relaxation.

Aravena *et al.* reported a new method for predicting effective demagnetization barriers (*U*_eff_) that considers all state energies and their contributions to the tunneling rate.^74–76^ The entire temperature range for complexes 1–3 can be divided into three regions (Fig. 7 and S33†). *U*_eff_ is always nearly zero in region I, since the contribution from the ground state dominates, while the ground state contribution drops in region II and KD_1_ becomes the first state to function; however, the dominating state changes to other higher excited KDs as the temperature continues to rise. *U*_eff_ grows to a constant value as the Orbach regime is approached as the temperature increases further in region III. KD_2–4_ are the three most important KD contributions to the *U*_eff_ values of complexes 1–3. We determined the *U*_eff_ values of complexes 1–3 using eqn (1)–(3); the saturated *U*_eff_ values for complexes 1–3 were calculated to be 159.3, 508.8 and 425.6 cm^−1^, respectively. Only when the relaxation temperatures exceed *ca.* 55 K for 1, *ca.* 60 K for 2 and *ca.* 50 K for 3, these saturations may be achieved. However, in reality, the highest experimental temperatures of the ac susceptibility signals for all complexes are below 50 K, thus leading to the calculated saturation *U*_eff_ being higher than the experimental values.

CF parameters *B* (*k*, *q*) with high percentages were calculated to further elucidate the mechanism of relaxation, the results of which are shown in Table S7.† The weight of the axial parameters *B* (2, 0) for 1 is very low, suggesting poor axial CF. However, for 2 and 3, the weights of absolute axial parameters *B* (2, 0) and *B* (4, 0) exceed 20% and 10%, respectively, and their values are both negative and larger than transverse *B* (*k*, *q*) (*k* = 2, 4; *q* ≠ 0), suggesting strong uniaxial anisotropy. Moreover, the value of the axial parameter *B* (2, 0) for complex 2 is larger than that of 3, which shows that 2 exhibits a larger CF than 3.

Taken together, the computational results are in complete accordance with the experimental observations. The high performance of SMM for 2 and 3 can be directly related to the strong axial nature of the ligand field arising from the short axial Dy–O bonds (2.108(5) Å for 2 and 2.087(3) Å for 3). Conversely, the SMM property of 1 is very poor, although the local symmetry of 1 is similar to that of 2 and 3. It is understandable that the long Dy–Cl bond generates the weak and spherical CF for 1, which may not be able to induce the strong magnetic anisotropy and afford a large *m*_J_ splitting. It is well known that the magnetic anisotropy and the local symmetry around the spin center seriously affect the properties of SMMs. The magnetic anisotropy determines the upper limit of the total CF splitting energy, while the high symmetry can suppress the QTM, resulting in the relaxation process in which the relaxation process passes through the higher excited KD. Therefore, a strong magnetic anisotropy is an essential precondition for high-performance SMMs. Despite the relatively low local symmetry around the Dy site, fascinating SMM behavior was observed for 2 and 3, which is ascribed to their strong, highly electrostatic Dy–O bond.

In addition, the apparent difference in the energy barrier for 2 and 3 is attributable to differences in the axial ligand. At first glance, complex 3 has a shorter axial Dy–O bond compared to 2; this should lead to a stronger axial ligand field acting on Dy(iii) for 3. However, this expectation contrasts with the fact that 2 possesses a better SMM property. Actually, the Dy(iii) electron density is oblate in shape, which requires the charge to be in the axial direction as much as possible. As reported previously,^35,41,42^ the introduction of electron-donating groups in the axial ligand improves the energy barrier. The LoProp charges in the ground KDs of 2 and 3 were calculated using the CASSCF wavefunction (Table S11†), with the axial oxygen atom in 2 found to be more charged than that in 3, which is ascribable to the superior electron-donating ability of the –CH_3_ group compared to the phenyl group. Therefore, the axial CH_3_O^−^ ligand improves SMM properties. Moreover, the charges on the axial oxygen atoms of both complexes are nearly three times larger than those on the neutral nitrogen atoms, which also indicates the axial nature of the total ligand field felt by the Dy(iii) ion in each case.

## Conclusion

The bulky heptadentate BPA-TPA ligand was successfully used to synthesize triangular dodecahedral dysprosium(iii)-based complexes with mono-axially ligated Cl^−^ (1), CH_3_O^−^ (2) or PhO^−^ (3) moieties. The large axial CF splitting of the *J* = 15/2 ground state induced by the short axial Dy–O bond results in slow magnetization relaxation through large anisotropic energy barriers (686 K for 2 and 469 K for 3). However, although the local coordination geometry of 1 was similar, only weak frequency-dependent ac signals without the *χ*_M_′′ maximum were observed for complex 1, which is caused by the weak and spherical CF generated by the long Dy–Cl bond. *Ab initio* calculations reveal that 2 and 3 exhibit dominant magnetization reversal barriers that expand to the second and first Kramers doublets, respectively.

According to theoretical predictions,^21^ the blocking barrier limit for a SIM is defined by a one-coordinate diatomic complex, such as [DyO]^+^, to be above 3000 K.^6^ However, the synthesis of a model compound with perfect axial symmetry is highly unrealistic and almost impossible to achieve. In the present case, the bulky pentapyridyldiamine ligand prevents additional coordination, and the methoxy or phenoxy oxygen atom provides strong CF splitting and forms a pseudo [DyO]^2+^ ligand field. This finding not only provides a promising blueprint for accessing linear mono-coordinate [DyO]^+^ complexes but also extends the kinds of high-performance SMMs available.

## Experimental section

### General procedures

All chemicals were commercially available and used without further purification. The [2,6-bis[bis(2-pyridylmethyl)amino]methyl]-pyridine (BPA-TPA) ligand was prepared as described previously.^59–61^ C, H and N elemental analyses were performed on an Elementar Vario EL III elemental analyzer. Powder XRD (PXRD) patterns were recorded at room temperature on a Bruker D8 Advance X-ray diffractometer (Fig. S1–S3†). Experimental PXRD patterns for bulk polycrystalline samples are consistent with those simulated from the single-crystal X-ray data, confirming the phase purity of complexes 1–3 and their stability in air. Single-crystal XRD data for 1–3 were collected at 296 K on a Bruker APEX II diffractometer equipped with a CCD area detector (Mo Kα radiation, *λ* = 0.71073 Å).^77,78^ All structures were solved using the SHELXTL-2016 program. Further crystallographic details are provided in Table S1 and Fig. S5–S7.† Complexes 1–3 were subjected to direct-current (dc) magnetic measurements between 2 and 300 K on a Quantum Design SQUID VSM magnetometer at fields up to 7 T. Alternating-current (ac) susceptibility measurements were carried out at ac frequencies in the 1–1000 Hz range in various applied static fields with an oscillating ac field of 2 Oe. Magnetic susceptibility data were corrected for diamagnetism associated with the constituent atoms, and the sample holder was estimated using Pascal constants.

### Synthesis of [Dy(BPA-TPA)Cl](BPh_4_)_2_ (1)

DyCl_3_·6H_2_O (0.188 g, 0.5 mmol), BPA-TPA (0.250 g, 0.5 mmol) and NaBPh_4_ (0.513 g, 1.5 mmol) were dissolved in methanol (20 mL), stirred for 30 min and then filtered, affording a white precipitate. White crystals suitable for single-crystal XRD were grown by the slow diffusion of diethyl ether into a CH_3_CN solution of the white precipitate over 2 d. Yield: 68% based on Dy(iii); elemental analysis (%) found (calcd) for C_79_H_71_B_2_ClDyN_7_: C, 70.86 (70.91); H, 5.34 (5.35); N, 7.30 (7.33).

### Synthesis of [Dy(BPA-TPA)(CH_3_O)](BPh_4_)_2_·CH_2_Cl_2_ (2)

DyCl_3_·6H_2_O (0.188 g, 0.5 mmol), BPA-TPA (0.250 g, 0.5 mmol) and sodium trimethylsilanolate (0.113 g, 1 mmol) were dissolved in methanol (15 mL) and refluxed for 4 h to give a yellow solution. NaBPh_4_ (0.343 g, 1 mmol) was then added to the yellow solution, and the reaction mixture was stirred for 30 min, at which time the product formed a light yellow precipitate. Yellow crystals suitable for single-crystal XRD were grown by the slow diffusion of hexane into a CH_2_Cl_2_ solution of the light yellow product over 2 d. Yield: 63% based on Dy(iii); elemental analysis (%) found (calcd) for C_81_H_76_B_2_Cl_2_DyN_7_O: C, 68.52 (68.58); H, 5.37 (5.40); N, 6.96 (6.91).

### Synthesis of [Dy(BPA-TPA)(OPh)](BPh_4_)_2_·2CH_2_Cl_2_ (3)

DyCl_3_·6H_2_O (0.188 g, 0.5 mmol) and BPA-TPA (0.250 g, 0.5 mmol) were dissolved in methanol (15 mL) and refluxed for 2 h. The solvent was removed under reduced pressure, and acetonitrile (10 mL) was added to the light yellow residue, after which phenol (0.0471 g, 0.5 mmol) and sodium trimethylsilanolate (0.0566 g, 0.5 mmol) were added to the resulting solution. The mixture was refluxed for 2 h and then filtered, and a solution of NaBPh_4_ (0.343 g, 1 mmol) in acetonitrile (10 mL) was added to the filtrate. After stirring for 30 min, the mixture was filtered, and the solvent was removed under vacuum to give the product as a yellow powder. Yellow crystals suitable for single-crystal XRD were grown by the slow diffusion of hexane into a CH_2_Cl_2_ solution of the light yellow product over 2 d. Yield: 59% based on Dy(iii); elemental analysis found (%) (calcd) for C_86_H_78_B_2_Cl_2_DyN_7_O: C, 69.74 (69.77); H, 5.28 (5.31); N, 6.66 (6.62).

### Computational details

CASSCF calculations on mononuclear complexes 1–3 (see Fig. 1 and S3† for the calculated complete structures of 1–3) based on single-crystal X-ray-determined geometries were performed using the OpenMolcas^70^ program package.

Atomic natural orbital basis sets from the MOLCAS ANO-RCC library were used: ANO-RCC-VTZP for Dy^III^; VTZ for close N and O; VDZ for distant atoms. The calculations used the second order Douglas–Kroll–Hess Hamiltonian, where scalar relativistic contractions were taken into account in the basis set, and spin–orbit couplings were handled separately in the restricted active space state interaction (RASSI-SO) procedure. For complexes 1–3, active electrons in seven active orbitals include all f electrons (CAS (9 in 7 for Dy^III^)) in the CASSCF calculation. To exclude all doubt, we calculated all roots in the active space. We mixed the maximum number of spin-free states that are possible using our hardware (all from 21 sextets, 128 from 224 quadruplets, 130 from 490 doublets for Dy^III^). The SINGLE_ANISO^71–73^ program was used to obtain energy levels, *g* tensors, magnetic axes (*etc.*), based on the above CASSCF/RASSI-SO calculations.

The theoretically predicted effective barrier has the following form as a function of temperature:^74,75^2
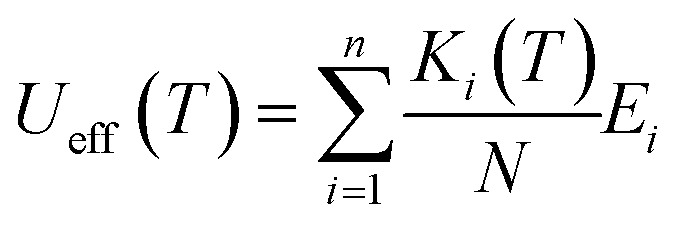
3
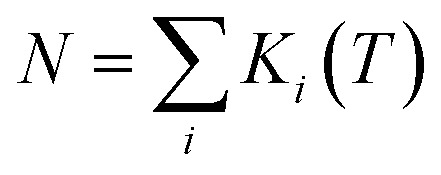


Each Kramers doublet (KD) has a particular demagnetization rate, namely:4
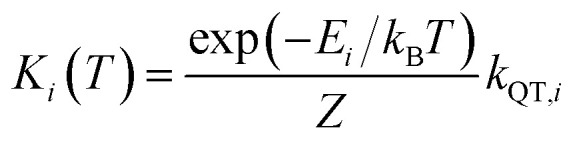
5
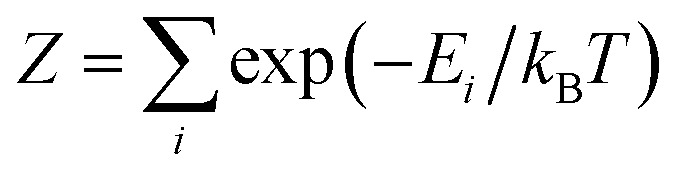
6
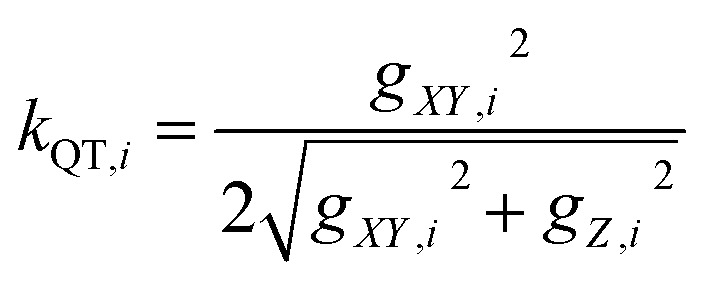
where *i* is the index for each KD, *E*_*i*_ represents the doublet energy obtained through CASSCF calculations, *Z* is the partition function, *k*_B_ is the Boltzmann constant, and *k*_QT,*i*_ is the tunneling relaxation rate for doublet *i*. The coefficient *K*_*i*_(*T*)/*N* that precedes *E*_*i*_ in eqn (2) represents the relative contribution of the corresponding KD to relaxation.

## Data availability

The datasets supporting this article have been uploaded as part of the ESI material.†

## Author contributions

L. C. supervised and conceived the project. B. Z. and Z. C. designed and performed the experiments. X. C. and R. J. performed the structural characterization and the data analysis. C. J. synthesized the ligands. Y.-Q. Z. developed the theoretical model. Y. W. performed the magnetic measurements. L. C. and Y.-Q. Z. wrote the paper. Z.-Y. L., A. Y., and H. K. reviewed and edited the manuscript. All coauthors discussed and commented on the manuscript.

## Conflicts of interest

There are no conflicts to declare.

## Supplementary Material

SC-013-D2SC03182E-s001

SC-013-D2SC03182E-s002
