# Supplementary material for: Pseudo-mono-axial ligand fields that support high energy barriers in triangular dodecahedral Dy(iii) single-ion magnets
Source: Chem Sci. 2022 Oct 31;13(44):13231–40. doi: 10.1039/d2sc03182e (PMC9667924; doi:10.1039/d2sc03182e)
Supplement: SC-013-D2SC03182E-s001 [file SC-013-D2SC03182E-s001.pdf]

Electronic Supplementary Material (ESI) for Chemical Science.
This journal is © The Royal Society of Chemistry 2022

Pseudo-mono-axial ligand fields that support high energy barriers in triangular dodecahedral Dy(III) single-ion magnets

Ben Zhang,‡[a] Zhijie Cheng,‡[a] Yingying Wu,[b] Lei Chen,*[a] Rong Jing,[a] Xingwei Cai,[a] Chunhui Jiang,[a] Yi-Quan Zhang,*[c] Aihua Yuan,*[a] Hui-Hui Cui[d] and Zhao-Yang Li*[b]

[a]*School of Environmental and Chemical Engineering, Jiangsu University of Science and Technology, Zhenjiang 212003, PR China.*

[b]*School of Materials Science and EngineeringNankai University, 38 Tongyan Road, Haihe Educational Park, Tianjin 300350, PR China.*

[c]*Jiangsu Key Laboratory for NSLSCS, School of Physical Science and Technology, Nanjing Normal University, Nanjing 210023, PR China.*

[d]*School of Chemistry and Chemical Engineering, Nantong University, Jiangsu 226019, PR China.*

Electronic Supplementary Information

Table of Contents

Crystal Data and Structures S3

Magnetic Characterization S9

Theory Calculation S25

Crystal Data and Structures

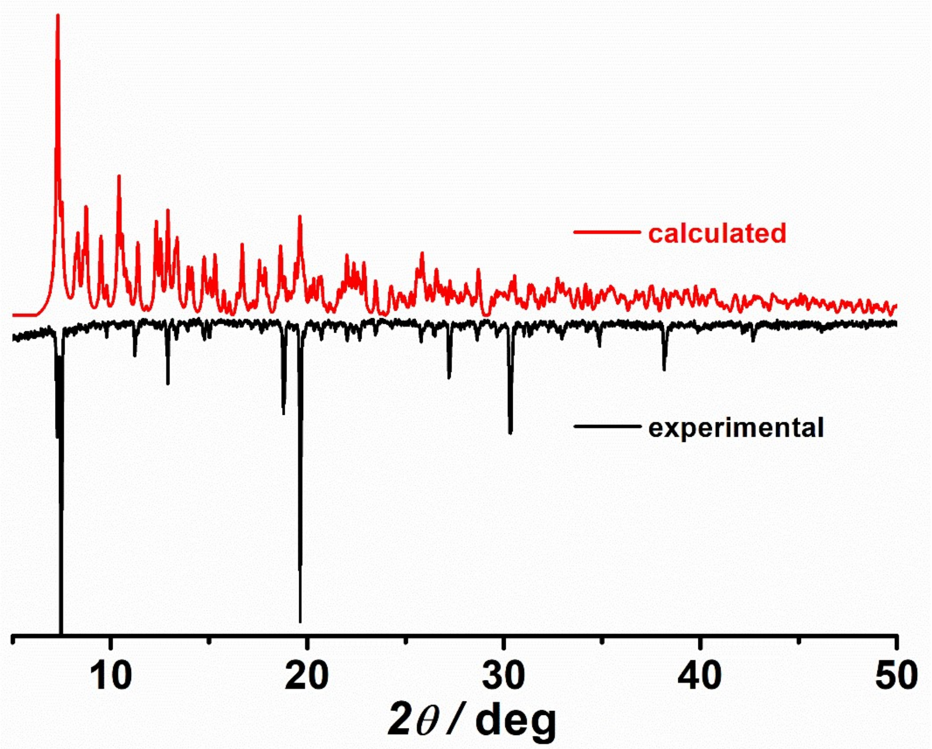

Figure S1. The XRD pattern for complex **1**.

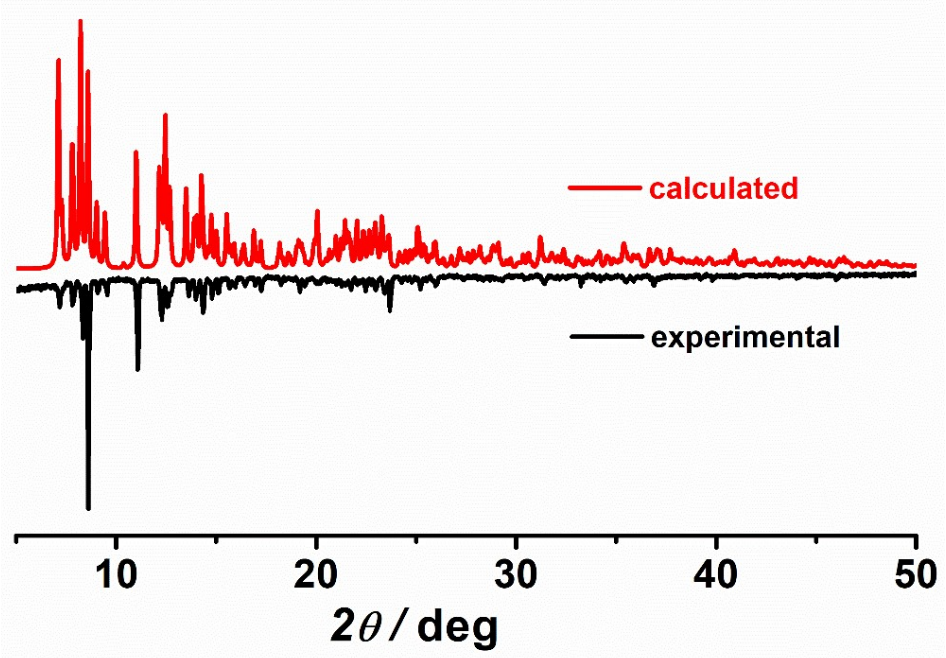

Figure S2. The XRD pattern for complex **2**.

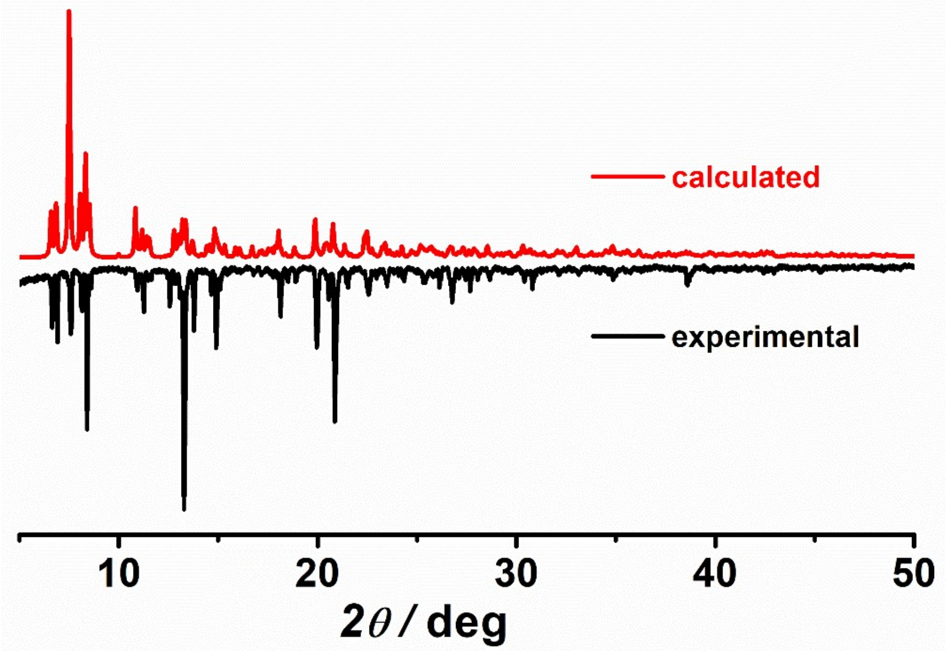

Figure S3. The XRD pattern for complex **3**.

Table S1. Crystal data and structure refinement for **1** and **2**.

	**1**	**2**	**3**
Molecular formula	$C_{79}H_{71}B_2ClDyN_7$	$C_{81}H_{76}B_2Cl_2DyN_7O$	$C_{86}H_{78}B_2Cl_2DyN_7O$
CCDC no	2212617	2166032	2166033
Formula weight	1337.99	1418.50	1480.57
Temperature/K	296(2)	296(2)	296(2)
Wavelength/Å	0.71073	0.71073	0.71073
crystal system	Monoclinic	Triclinic	Triclinic
Space group	*P*21/c	$P\bar{1}$	$P\bar{1}$
a/Å	23.6641(7)	12.7371(3)	14.0388(2)
b/Å	14.1103(4)	14.2679(4)	14.4810(2)
c/Å	21.3612(6)	22.1844(6)	21.4518(3)
α/deg	90	101.261(2)	91.2700(10)
β/deg	96.266(2)	93.525(2)	97.2790(10)
γ/deg	90	116.025(2)	111.8680(10)
V/Å^3	7090.1(4)	3503.18(17)	4003.23(10)
Z	4	2	2
D_{calc}, Mg/m^3	1.253	1.345	1.228
μ/mm^{-1}	1.139	1.194	1.048
F (000)	2748	1458	1522
Goodness-of-fit on F^2	0.986	0.897	1.075
Final R indices [$I > 2\sigma(I)$][a]	R1=0.0598, wR2=0.0697	R1=0.0573, wR2= 0.1483	R1=0.0470, wR2=0.1071
R indices (all data)[a]	R1=0.1654, wR2=0.0842	R1=0.0913, wR2=0.1688	R1=0.0707, wR2=0.1173

[a] $wR_2 = [\Sigma[w(F_o^2–F_c^2)^2]/\Sigma[w(Fo^2)^2]]^{1/2}$, $R_1 = \Sigma||F_o|–|F_c||/\Sigma|F_o|$.

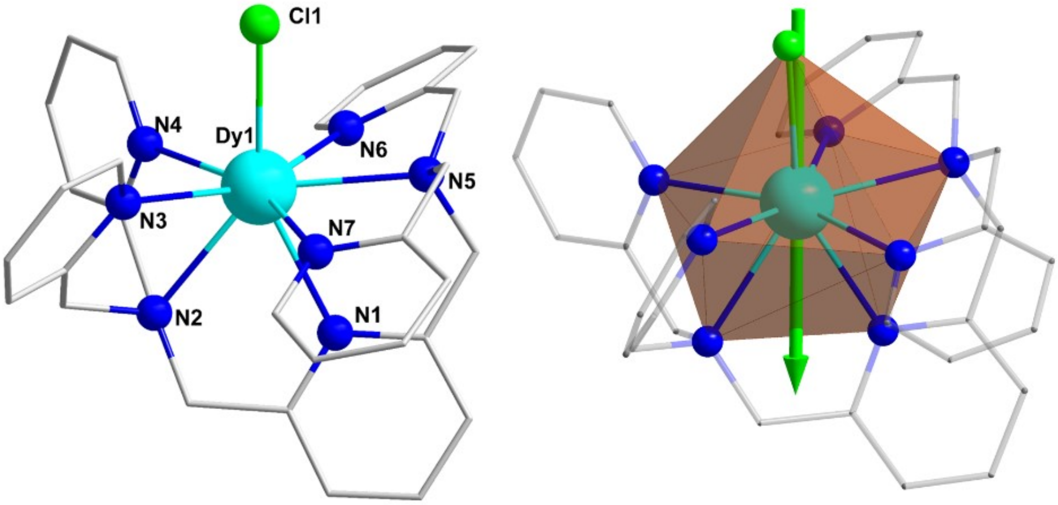

Figure S4. Coordinate structure and calculated orientation of the local main magnetic axes on Dy^{III} ions in the ground KDs of $[Dy(BPA\text{-}TPA)(X)]^{2+}$ for complexes **1**. Color scheme: Dy, cyan; N, blue; Cl, green; C, gray. H-atoms have been omitted for clarity.

Table S2. Selected bond lengths (Å) for **1**.

	**1**
**Dy(1)-Cl(1)**	**2.5835(16)**
Dy(1)-N(1)	2.489(4)
Dy(1)-N(2)	2.539(4)
Dy(1)-N(3)	2.525(5)
Dy(1)-N(4)	2.458(5)
Dy(1)-N(5)	2.515(4)
Dy(1)-N(6)	2.619(5)
Dy(1)-N(7)	2.541(5)

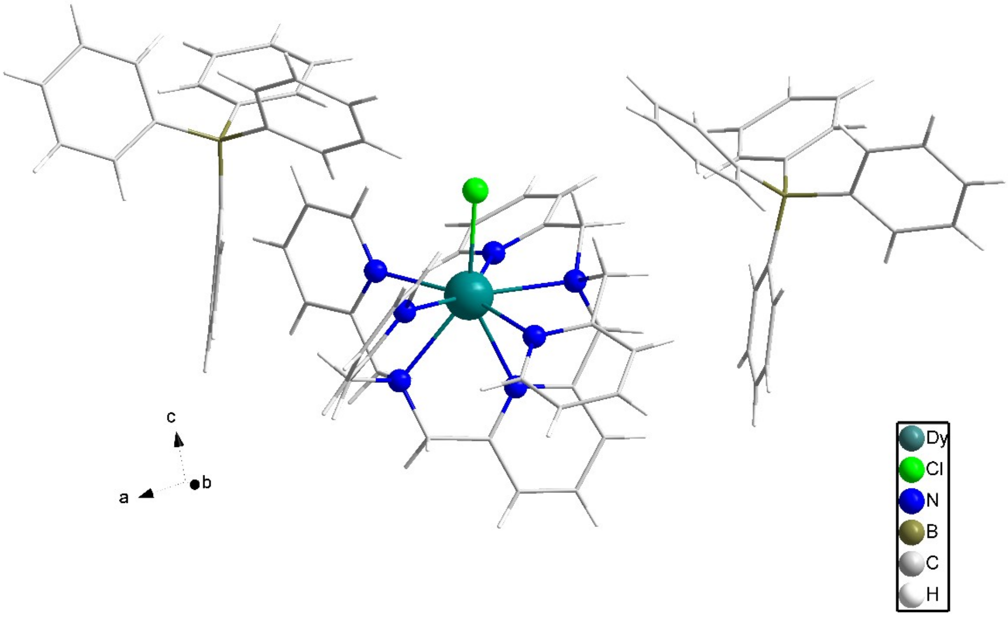

Figure S5. View of the molecular structure of **1**.

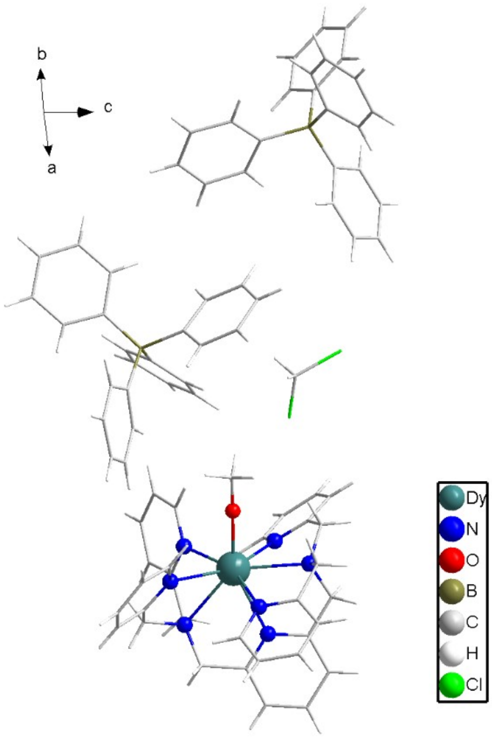

Figure S6. View of the molecular structure of **2**.

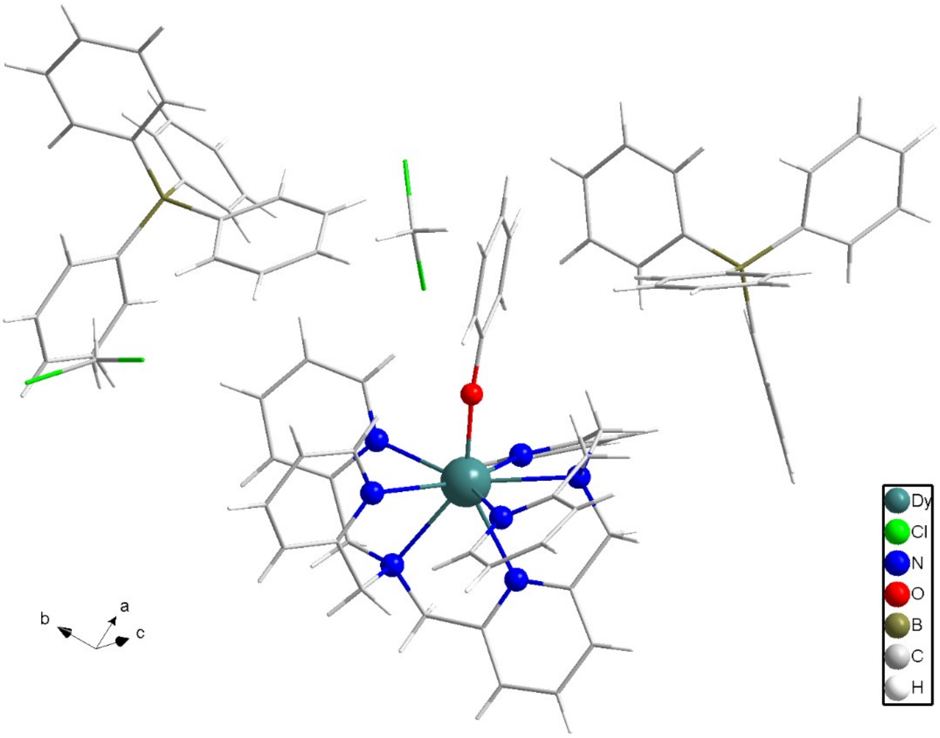

Figure S7. View of the molecular structure of **3**.

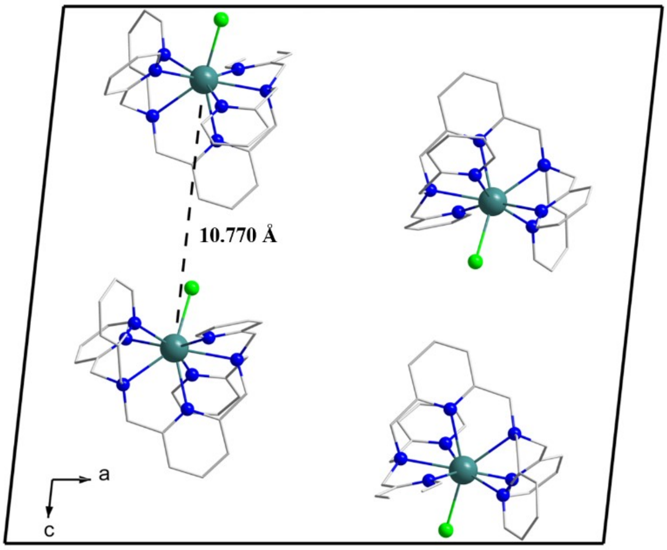

Figure S8. Stacking between adjacent complexes in **1**. The BPh_4^- anions and H atoms are omitted for clarity.

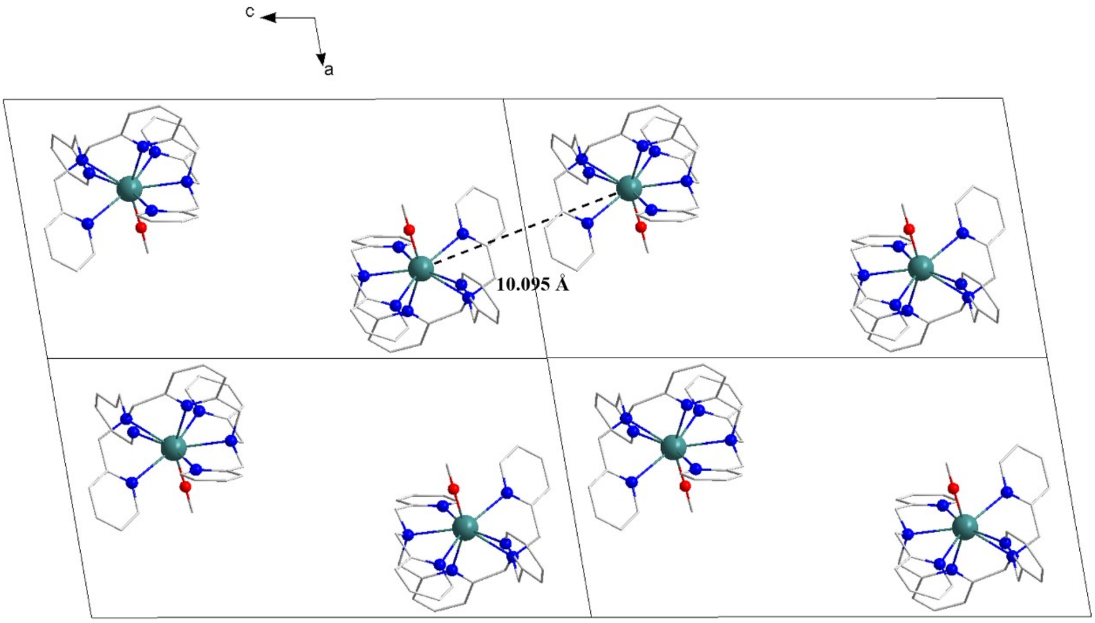

Figure S9. Stacking between adjacent complexes in **2**. The BPh_4^- anions, CH_2Cl_2 molecules and H atoms are omitted for clarity.

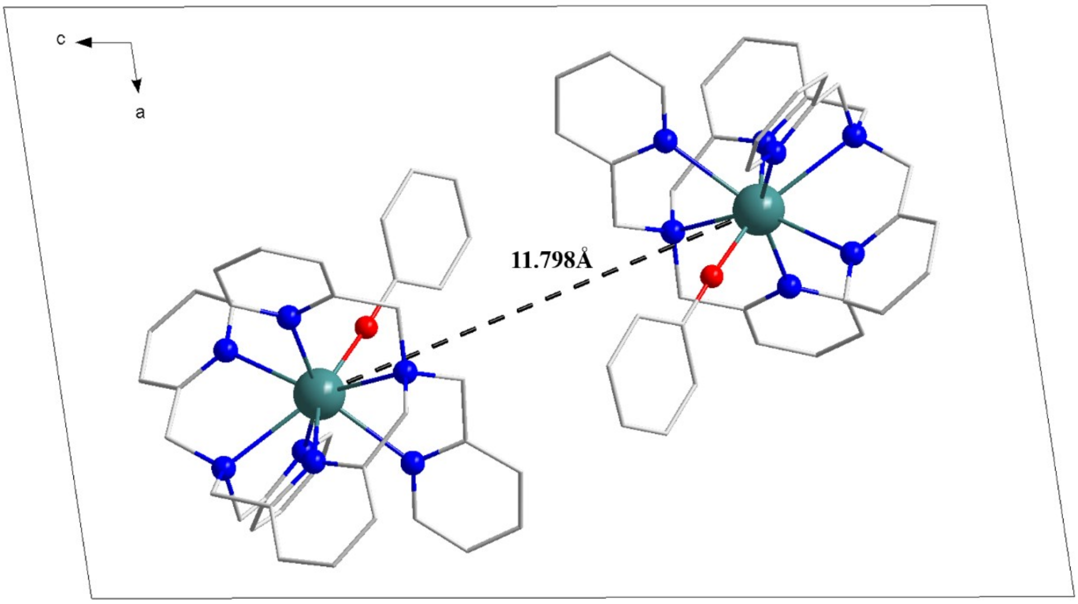

Figure S10. Stacking between adjacent complexes in **3**. The BPh_4^- anions, CH_2Cl_2 molecules and H atoms are omitted for clarity.

Table S3. Continuous Shape Measure (CShM) analyses for **1**−**3**. The lowest CShM value is highlighted.

Ideal Polyhedron	**1**	**2**	**3**
Octagon (D_{8h})	31.992	32.230	31.614
Heptagonal pyramid (C_{7v})	22.384	21.942	22.984
Hexagonal bipyramid (D_{6h})	11.027	10.516	10.047
Cube (O_h)	9.110	8.248	10.738
Square antiprism (D_{4d})	4.406	4.443	3.935
Triangular dodecahedron (D_{2d})	**2.166**	**2.130**	**1.820**
Johnson gyrobifastigium (D_{2d})	10.738	10.218	12.019
Johnson elongated triangular bipyramid (D_{3h})	26.889	25.946	24.240
Biaugmented trigonal prism J50 (C_{2v})	4.199	3.826	2.466
Biaugmented trigonal prism (C_{2v})	3.297	3.369	2.228
Snub diphenoid (D_{2d})	5.769	5.367	3.873
Triakis tetrahedron (T_d)	9.480	8.962	11.575
Elongated trigonal bipyramid (D_{3h})	22.623	22.412	20.964

Magnetic Characterization

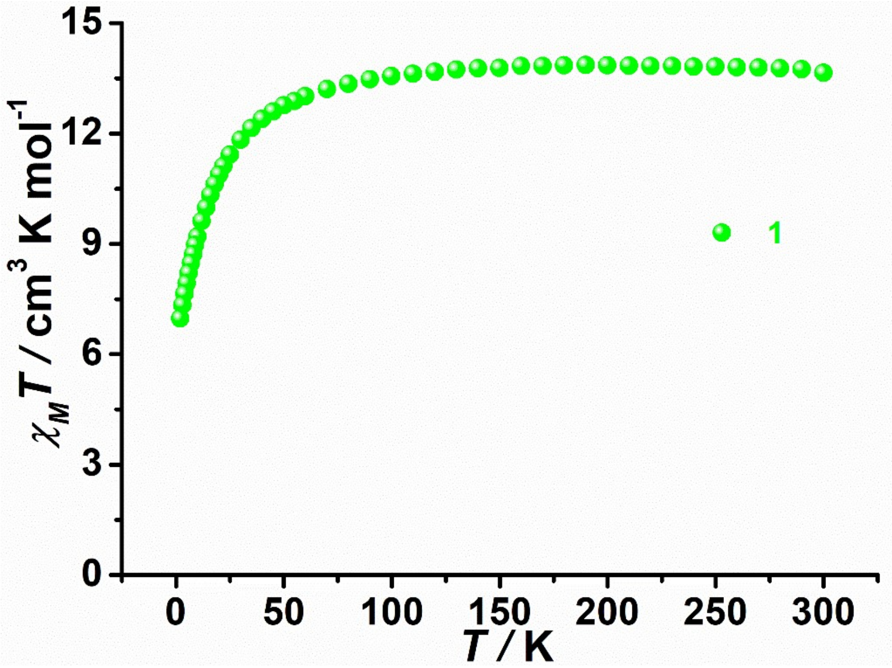

Figure S11. Variable-temperature dc susceptibility data for **1** in a 1000 Oe applied dc field.

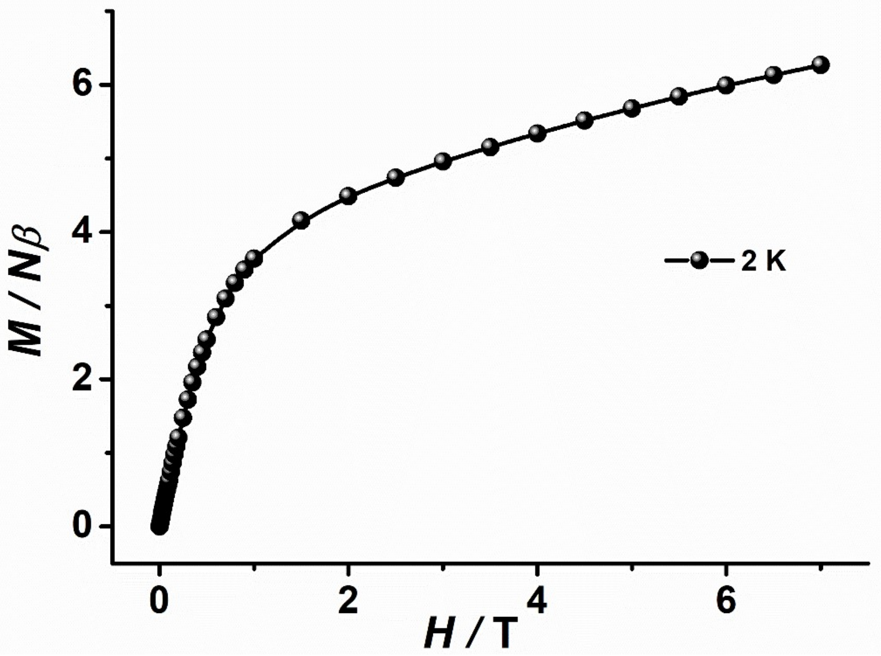

Figure S12. The field-dependence of magnetization at 2 K for **1**. The solid lines are for eye guidance.

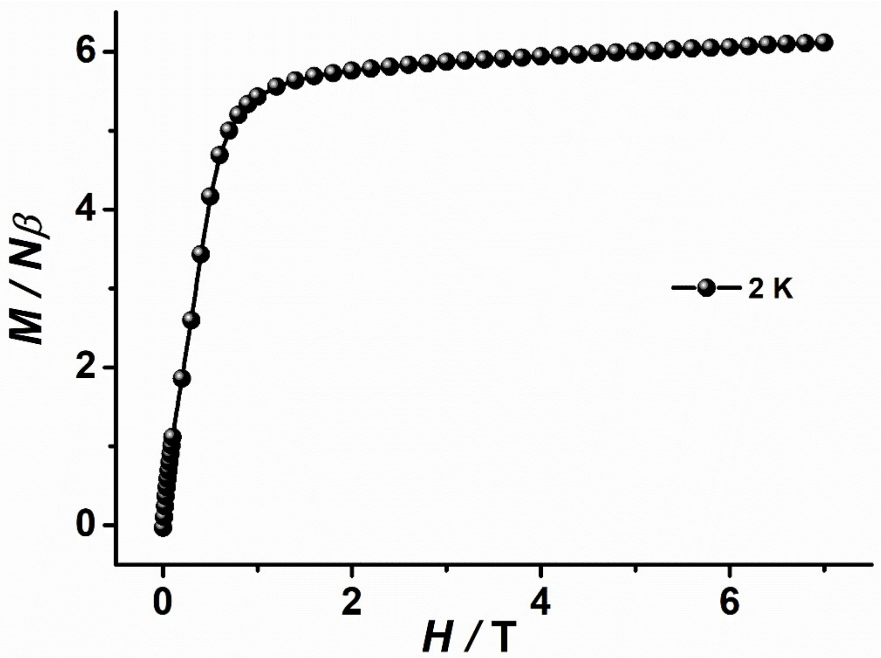

Figure S13. The field-dependence of magnetization at 2 K for 2. The solid lines are for eye guidance.

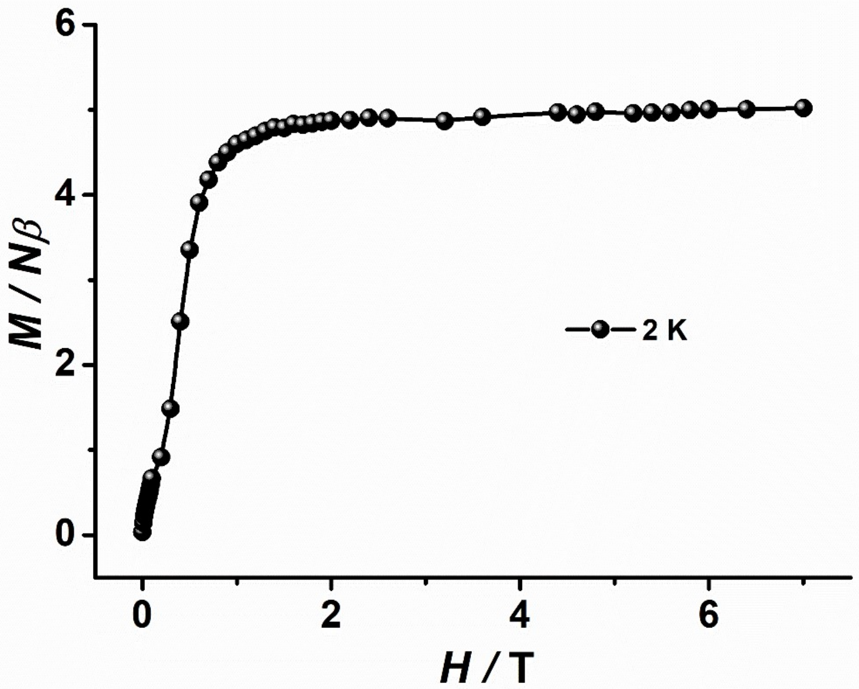

Figure S14. The field-dependence of magnetization at 2 K for 2. The solid lines are for eye guidance.

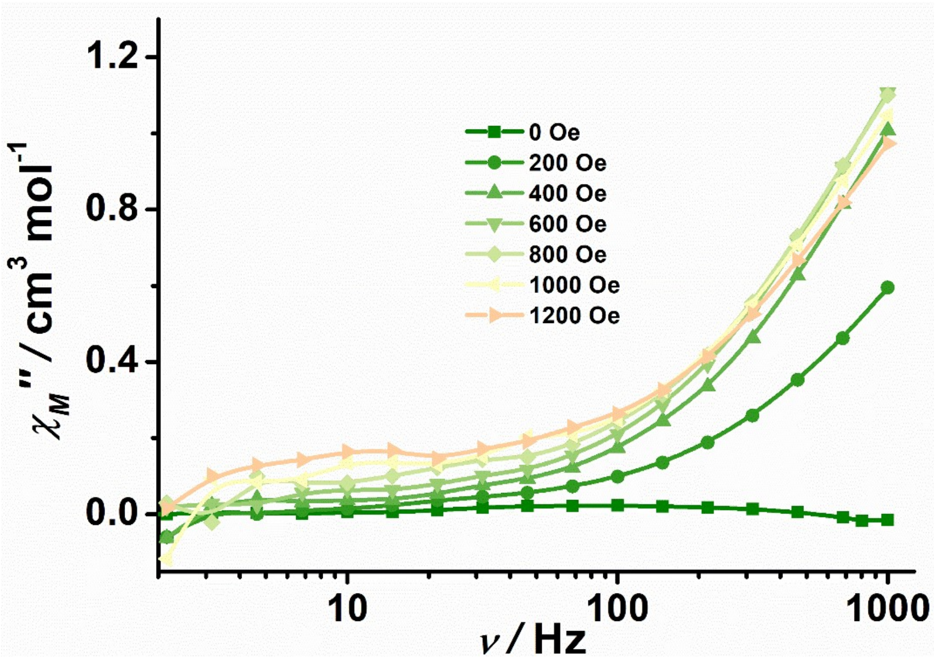

Figure S15. Field dependence of out-of-phase ac susceptibility (χ_M'') for **1** at 2 K. The lines are guides for the eye.

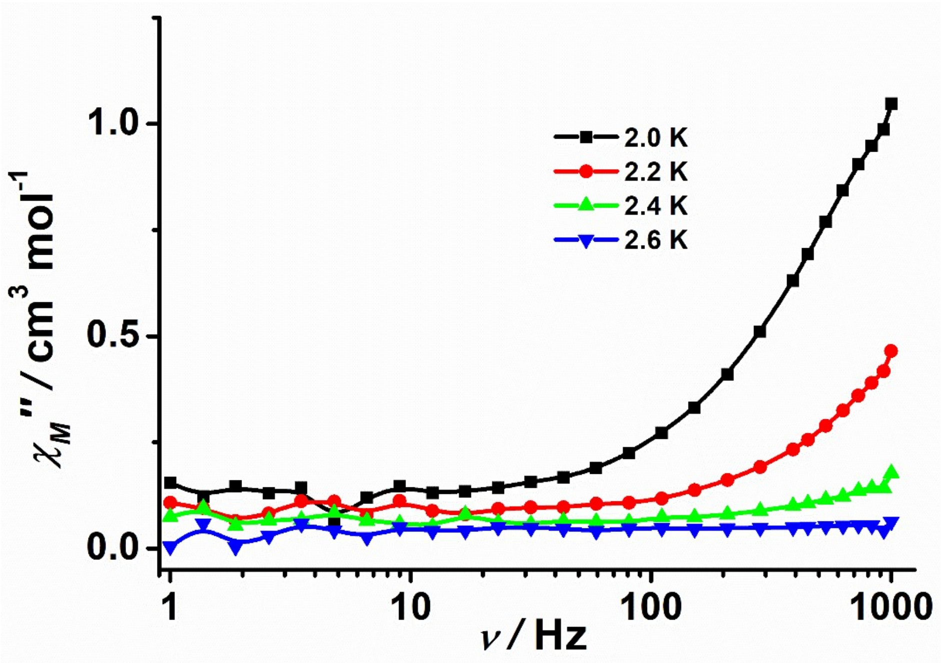

Figure S16. Frequency dependence of in-phase (χ_M') and out-of-phase ac susceptibility (χ_M'') for **1** under 800 Oe dc field. The lines are guides for the eye.

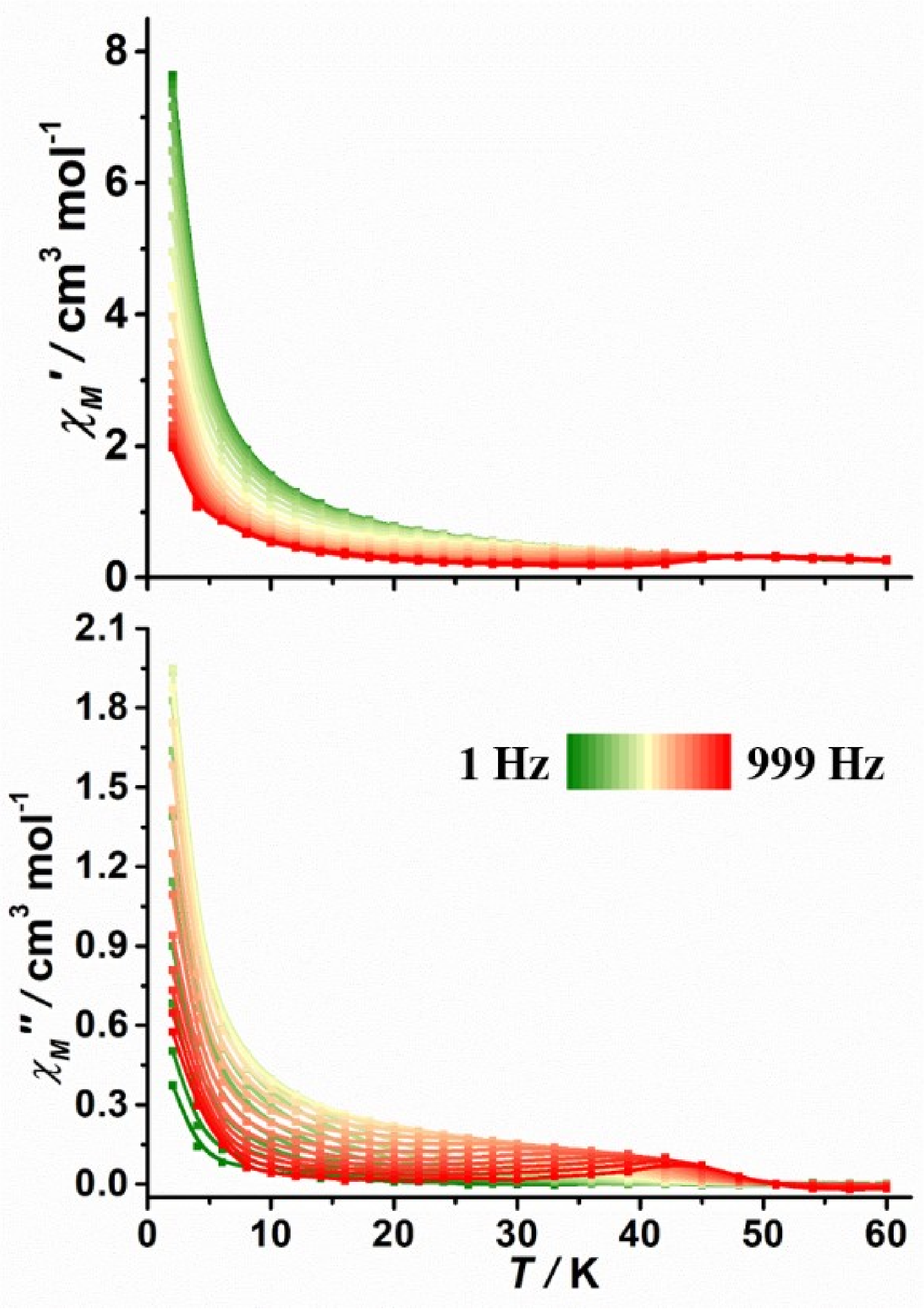

Figure S17. Temperature dependence of in-phase (χ_M') and out-of-phase ac susceptibility (χ_M'') for **2** under zero dc field; the solid lines are guides for the eye.

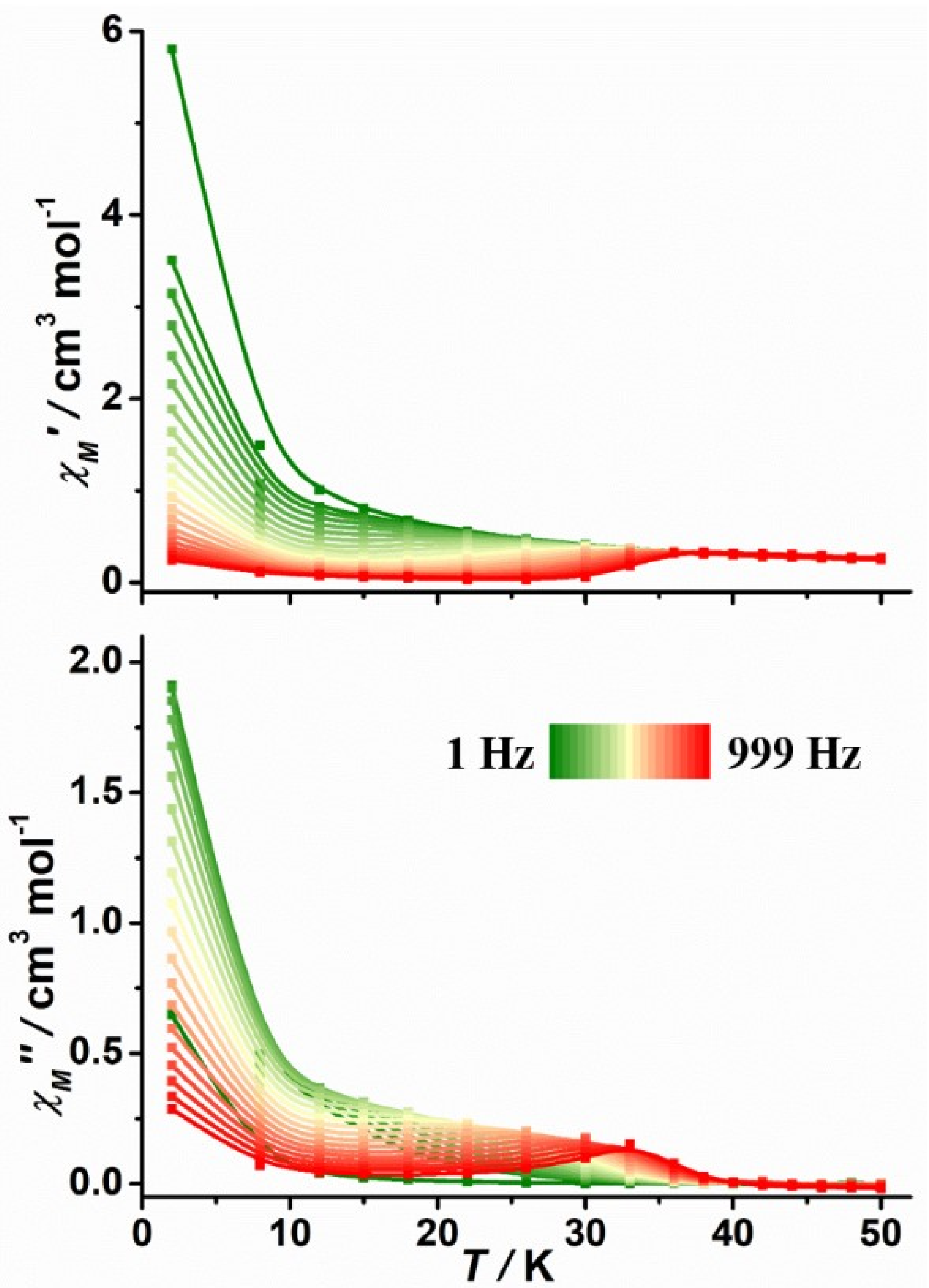

Figure S18. Temperature dependence of in-phase (χ_M') and out-of-phase ac susceptibility (χ_M'') for **3** under zero dc field; the solid lines are guides for the eye.

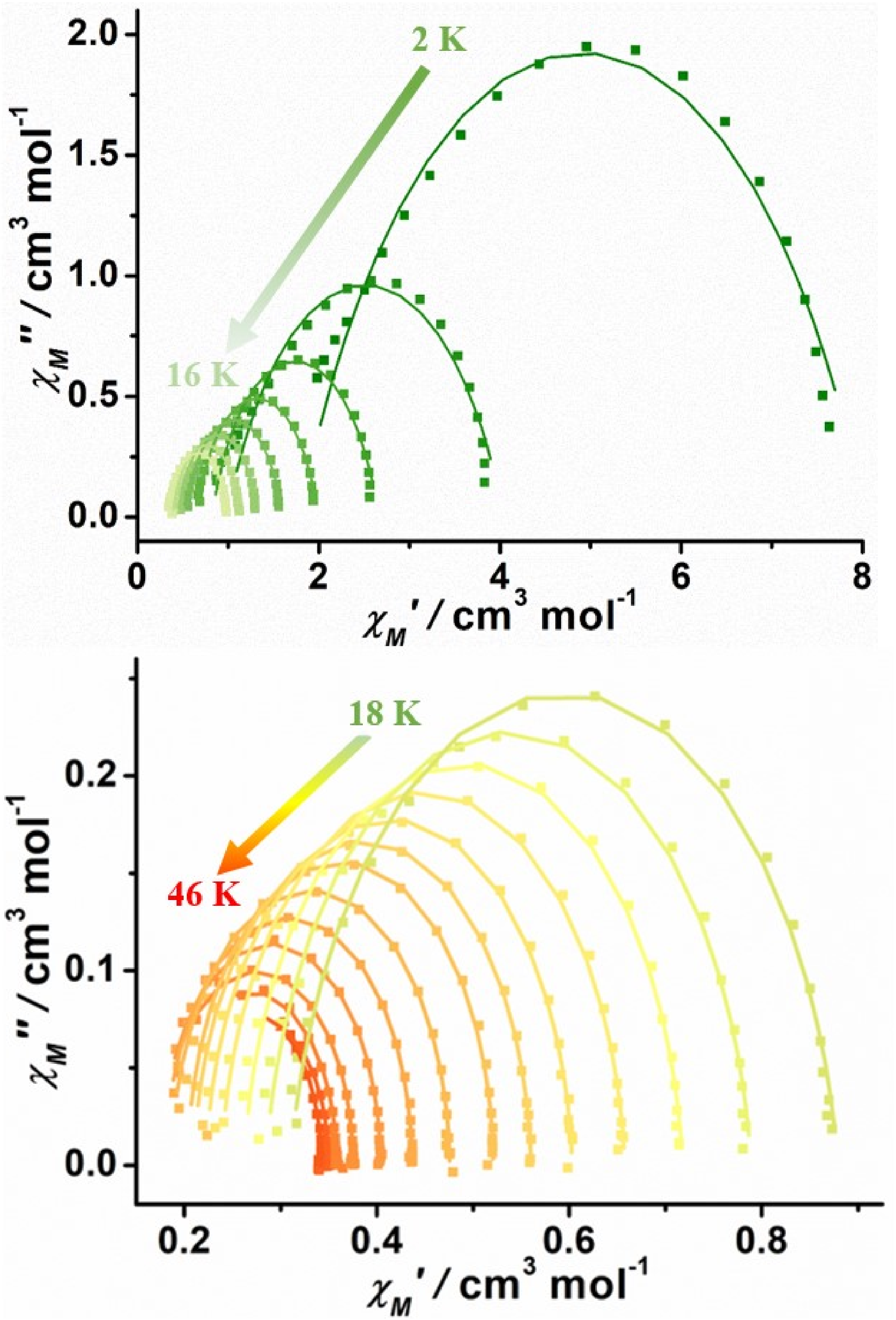

Figure S19. Cole-Cole plot of **2** under zero dc field, the solid lines correspond to the best fit to Debye's law.

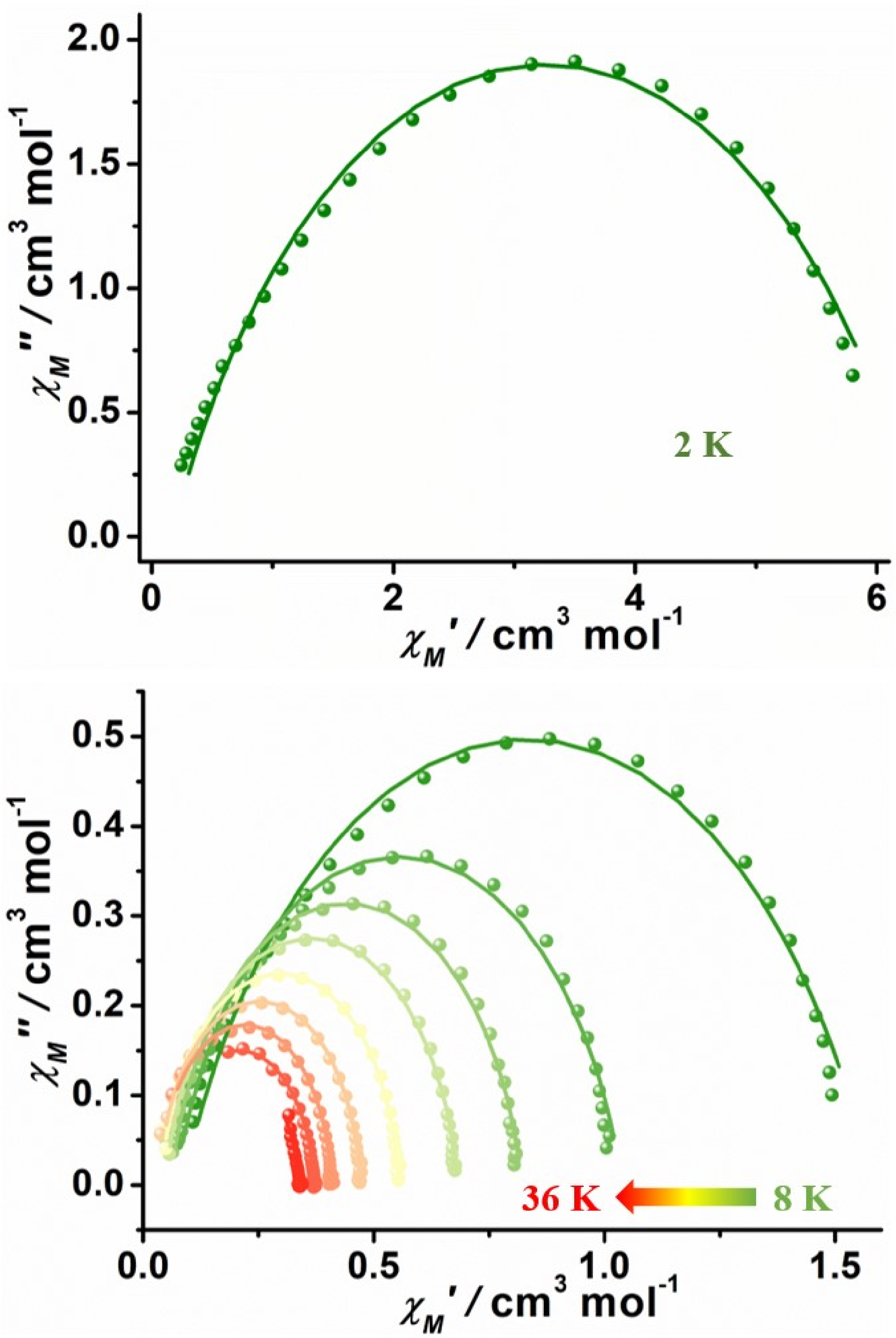

Figure S20. Cole-Cole plot of **3** under zero dc field, the solid lines correspond to the best fit to Debye's law.

Table S4. The parameters obtained by fitting the Cole-Cole plot under zero dc field for **2**.

T/K	χ_S	χ_T	τ	a
2	1.80	8.02	0.00646	0.29
4	0.99	4.04	0.00603	0.28
6	0.82	2.66	0.00639	0.22
8	0.65	1.99	0.00635	0.19
10	0.53	1.59	0.0061	0.17
12	0.45	1.32	0.00574	0.15
14	0.39	1.14	0.0053	0.14
16	0.35	0.99	0.00483	0.11
18	0.31	0.88	0.00425	0.10
20	0.28	0.79	0.00371	0.08
22	0.26	0.72	0.00321	0.06
24	0.24	0.66	0.0027	0.06
26	0.22	0.60	0.0023	0.04
28	0.21	0.56	0.00193	0.03
30	0.20	0.52	0.00163	0.01
33	0.19	0.49	0.00122	0.01
36	0.18	0.44	9.03601×10^{-4}	~0
39	0.18	0.40	6.16083×10^{-4}	~0
42	0.18	0.38	3.32957×10^{-4}	~0
43	0.19	0.37	2.60757×10^{-4}	~0
44	0.18	0.36	1.91021×10^{-4}	~0
45	0.20	0.35	1.38264×10^{-4}	~0
46	0.18	0.34	9.11627×10^{-5}	~0

Table S5. The parameters obtained by fitting the Cole-Cole plot under zero dc field for **3**.

T/K	χ_S	χ_T	τ	a
2	0.16	6.35	0.01204	0.30
8	0.09	1.58	0.00775	0.25
12	0.07	1.04	0.0057	0.18
15	0.06	0.82	0.0041	0.12
18	0.05	0.68	0.00287	0.09
22	0.04	0.55	0.00175	0.05
26	0.03	0.47	0.00106	0.04
30	0.04	0.41	5.01966×10^{-4}	0.02
33	0.05	0.37	1.87543×10^{-4}	0.05
36	~0	0.34	3.59928×10^{-5}	0.10

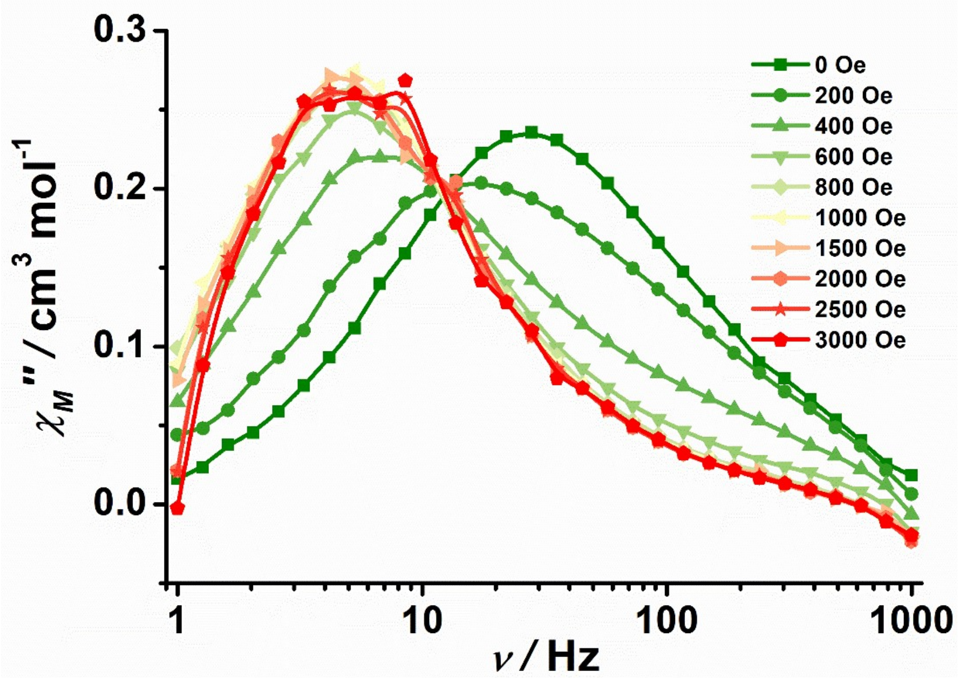

Figure S21. Field dependence of out-of-phase ac susceptibility (χ_M'') for **2** at 15 K. The lines are guides for the eye.

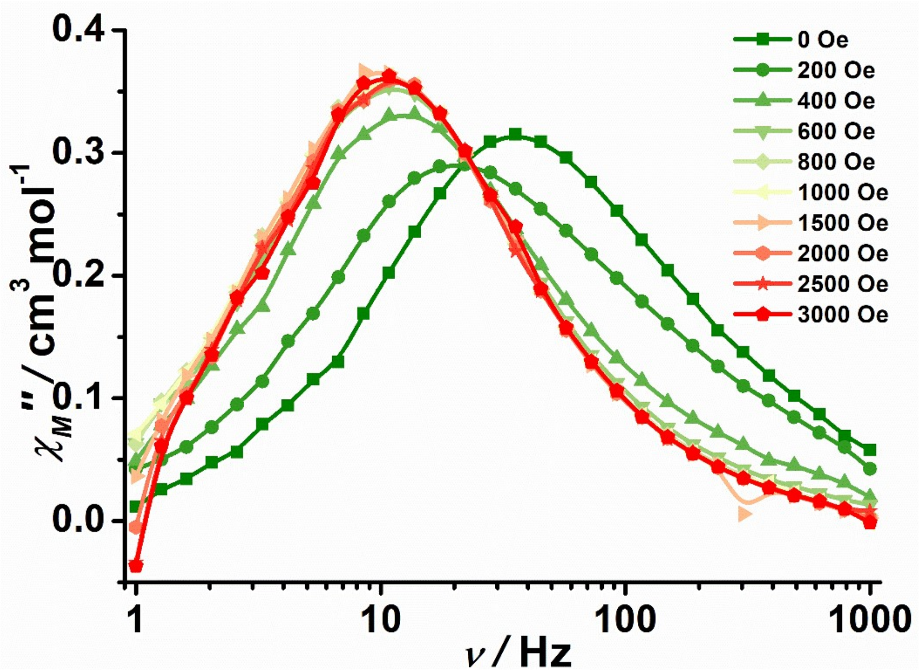

Figure S22. Field dependence of out-of-phase ac susceptibility (χ_M'') for **3** at 15 K. The lines are guides for the eye.

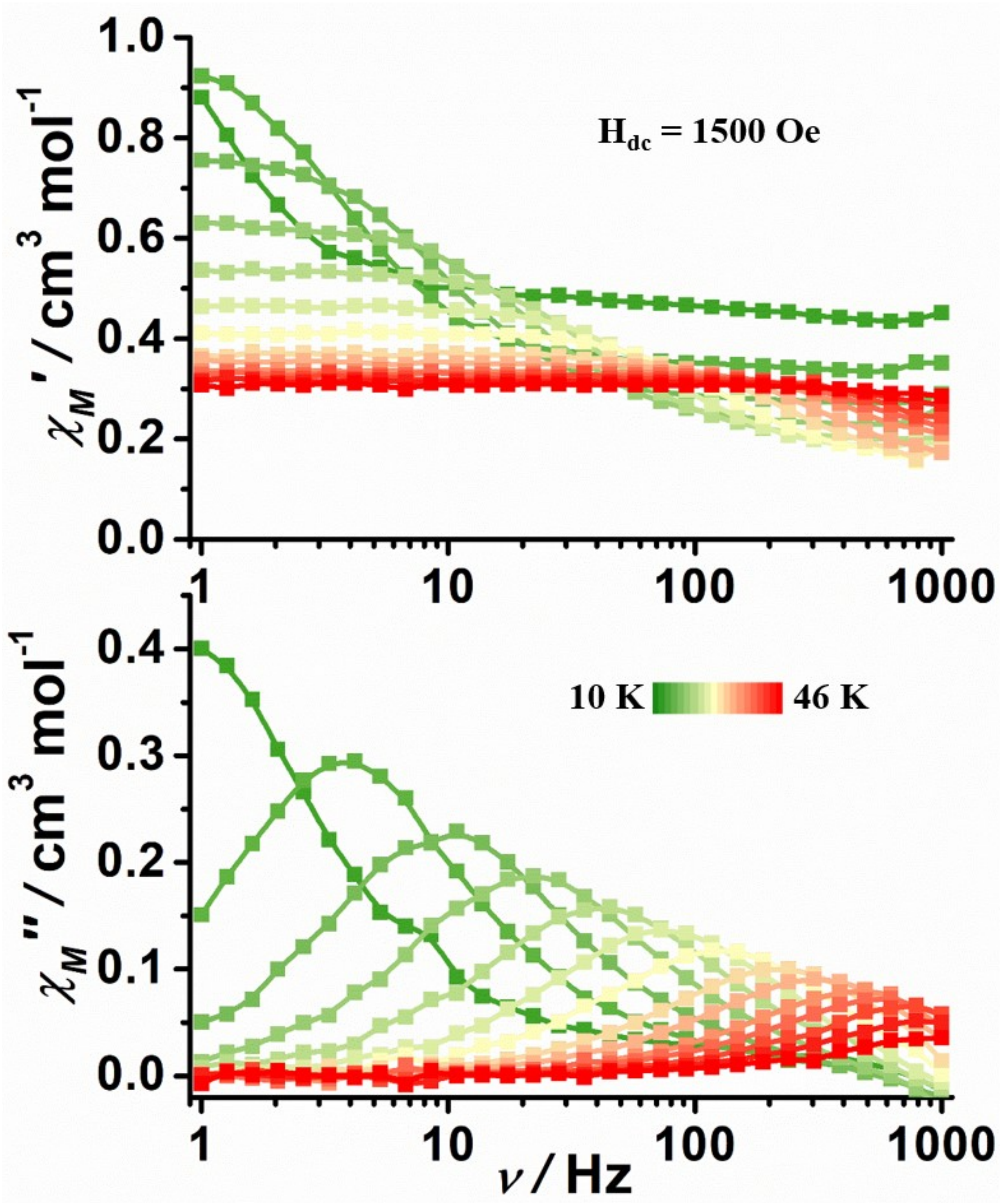

Figure S23. Frequency dependence of in-phase (χ_M') and out-of-phase ac susceptibility (χ_M'') for **2** under 1500 Oe dc field. The lines are guides for the eye.

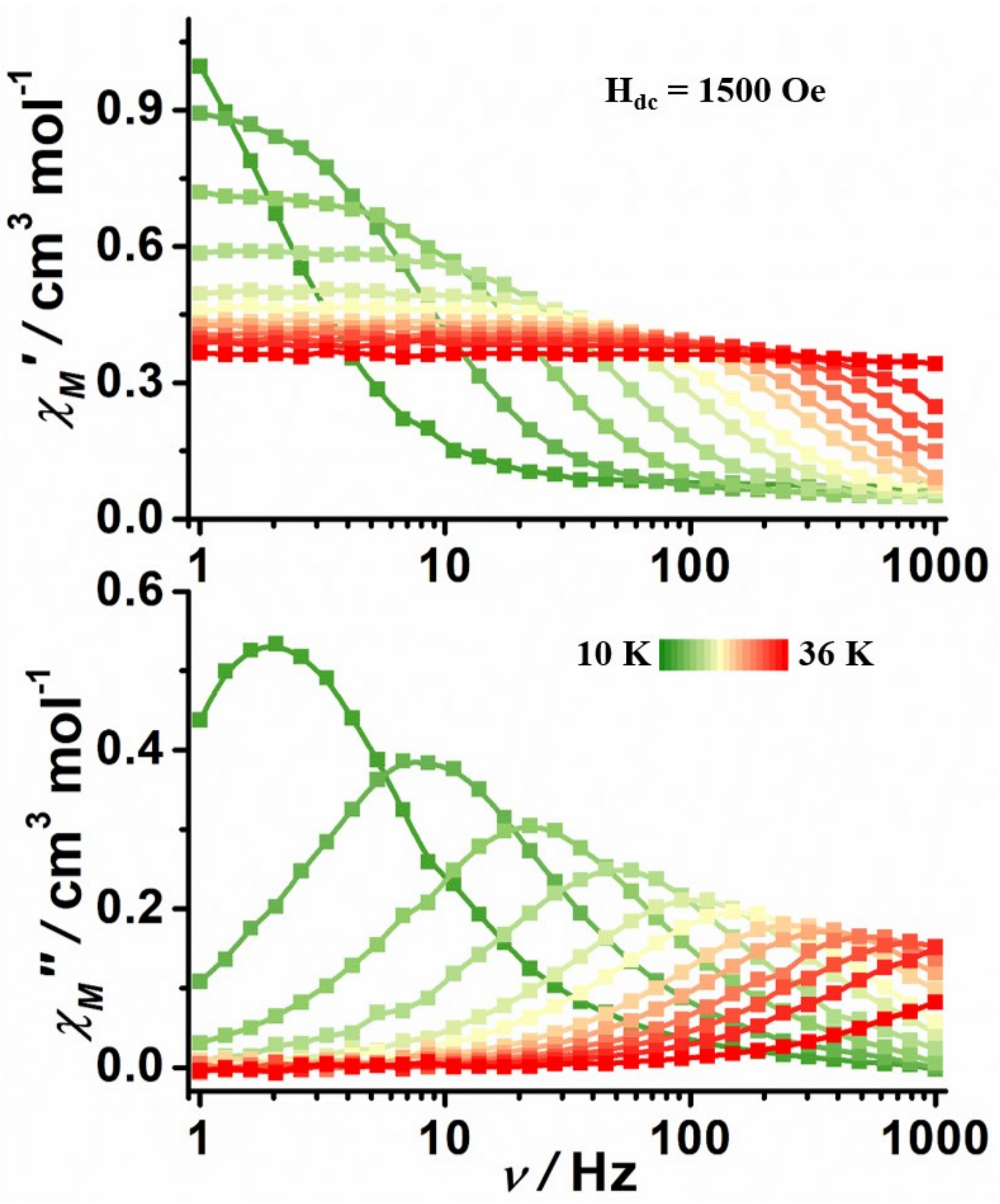

Figure S24. Frequency dependence of in-phase (χ_M') and out-of-phase ac susceptibility (χ_M'') for **3** under 1500 Oe dc field. The lines are guides for the eye.

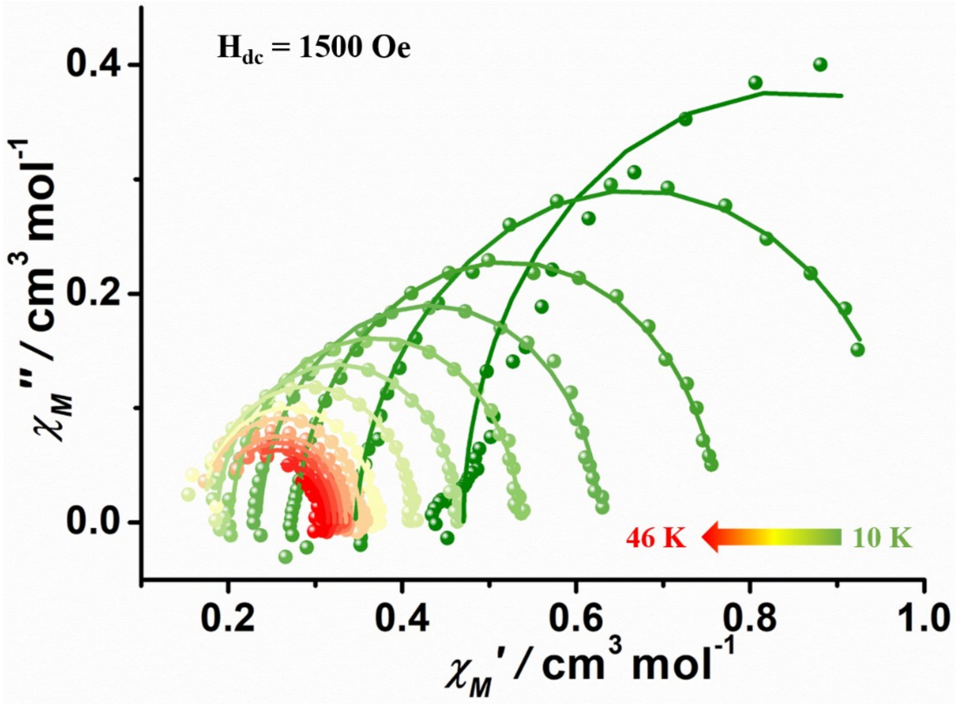

Figure S25. Cole-Cole plot of **2** under 1500 Oe dc field, the solid lines correspond to the best fit to Debye's law.

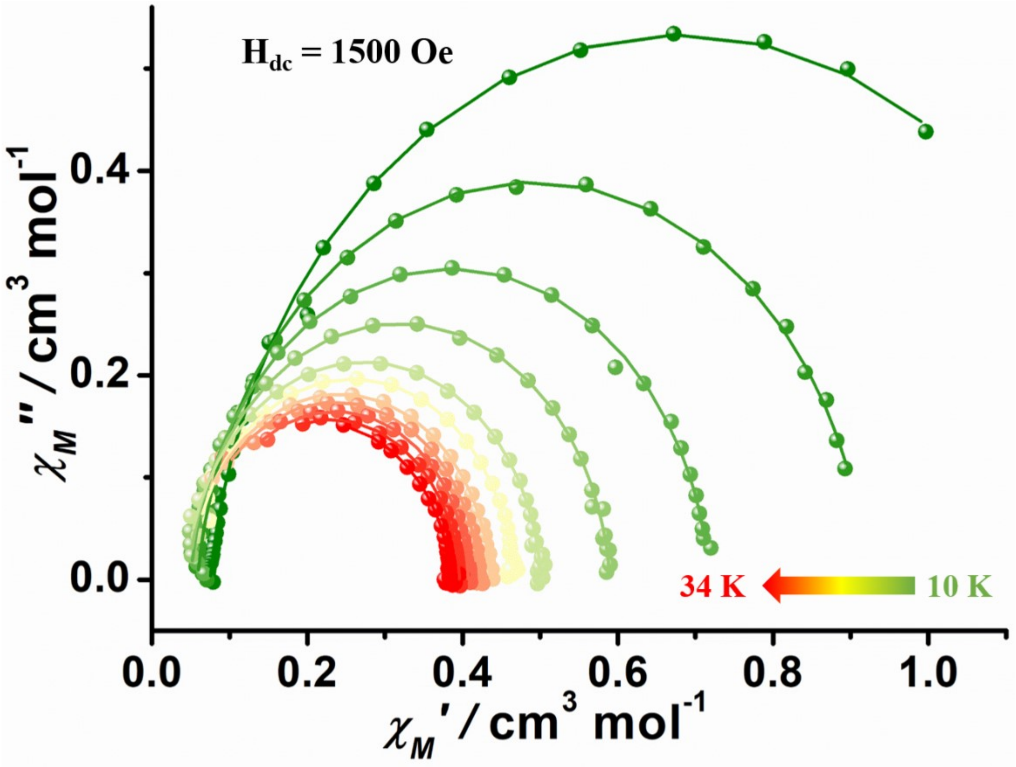

Figure S26. Cole-Cole plot of **3** under 1500 Oe dc field, the solid lines correspond to the best fit to Debye's law.

Table S6. The parameters obtained by fitting the Cole-Cole plot under 1500 Oe dc field for **2**.

T/K	χ_S	χ_T	τ	a
10	0.47	1.23	0.13725	~0
14	0.35	0.99	0.04162	0.06298
18	0.28	0.76	0.01592	0.04215
22	0.24	0.63	0.00728	0.01781
26	0.20	0.53	0.00381	0.01465
28	0.19	0.46	0.00217	~0
32	0.17	0.41	0.0013	~0
36	0.18	0.37	7.66876×10^{-4}	~0
40	0.17	0.35	5.38432×10^{-4}	~0
42	0.18	0.34	3.58324×10^{-4}	~0
43	0.19	0.33	2.8197×10^{-4}	~0
44	0.20	0.32	2.12894×10^{-4}	~0
45	0.21	0.31	1.64189×10^{-4}	~0
46	0.23	0.31	1.17752×10^{-4}	~0

Table S7. The parameters obtained by fitting the Cole-Cole plot under 1500 Oe dc field for **3**.

T/K	χ_S	χ_T	τ	a
10	0.08	1.29	0.07923	0.08
14	0.06	0.92	0.01936	0.07
18	0.05	0.72	0.00714	0.06
22	0.05	0.59	0.00319	0.05
26	0.04	0.50	0.00154	0.04
28	0.05	0.46	0.00104	0.03
30	0.05	0.44	6.04704×10^{-4}	0.04
31	0.05	0.42	4.3747×10^{-4}	0.04
32	0.06	0.41	3.09546×10^{-4}	0.02
33	0.06	0.40	2.10486×10^{-4}	0.04
34	0.05	0.39	1.25063×10^{-4}	0.06

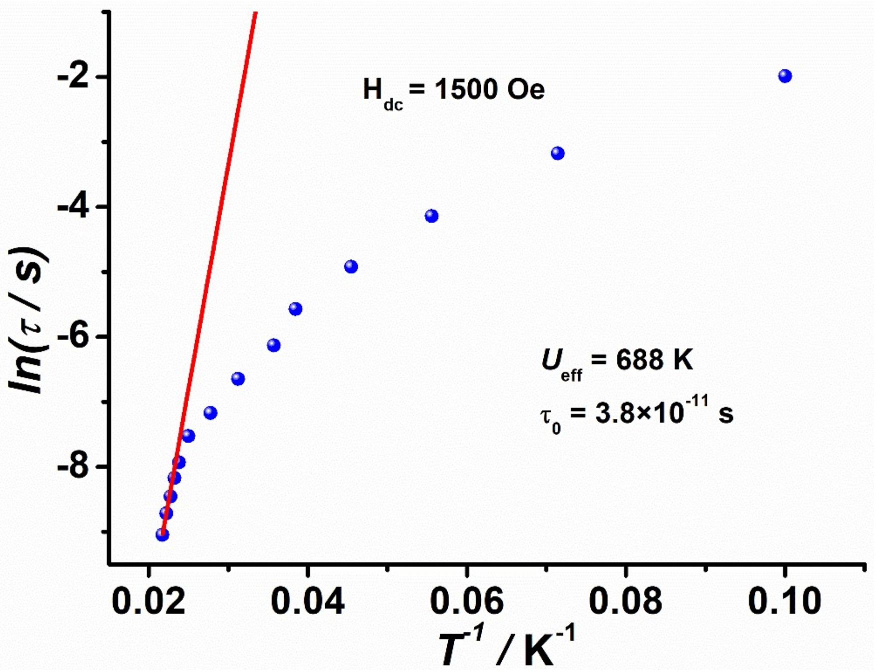

Figure S27. Plot of *ln(τ)* as functions of T^{-1} under 1500 Oe dc field for **2**. The red line corresponds to the Arrhenius law at high temperatures.

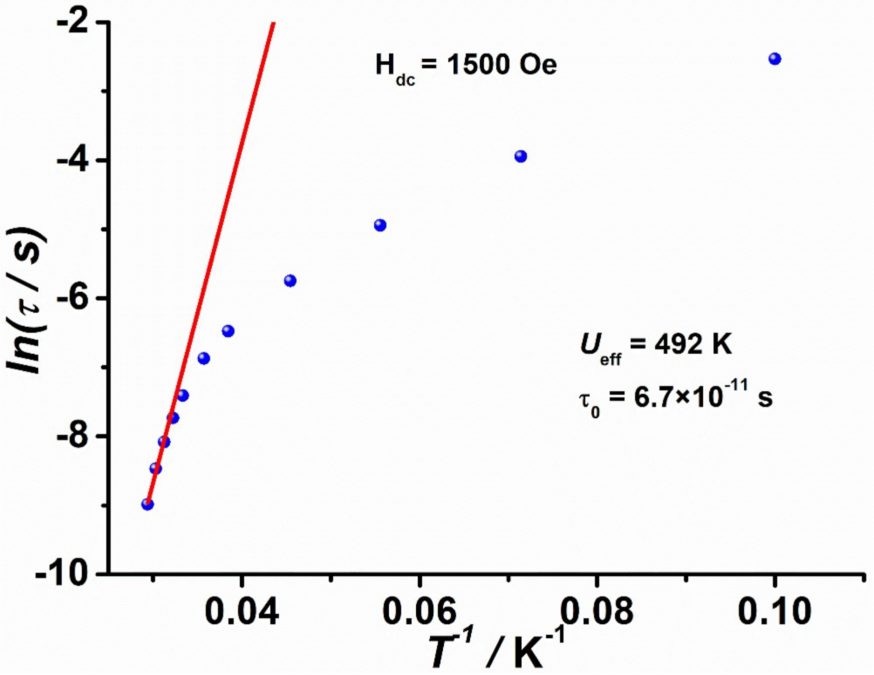

Figure S28. Plot of *ln(τ)* as functions of T^{-1} under 1500 Oe dc field for **3**. The red line corresponds to the Arrhenius law at high temperatures.

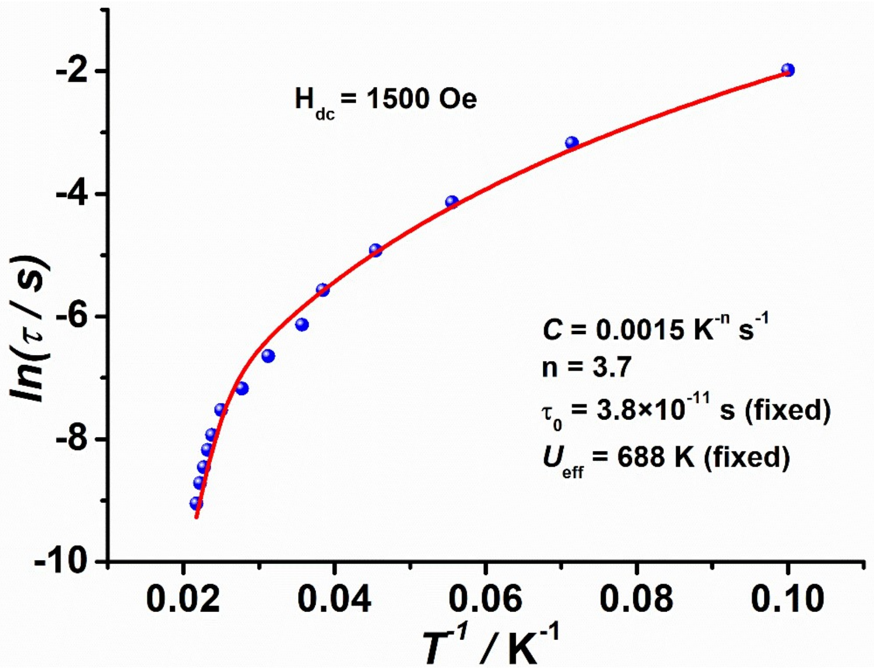

Figure S29. Plot of *ln(τ)* as functions of T^{-1} under 1500 Oe dc field for **2**. The green solid line is the best fit using the combination of Raman and Orbach processes based on the fixed values of U_{eff} = 688 K and $\tau_0 = 3.8 \times 10^{-11}$ s.

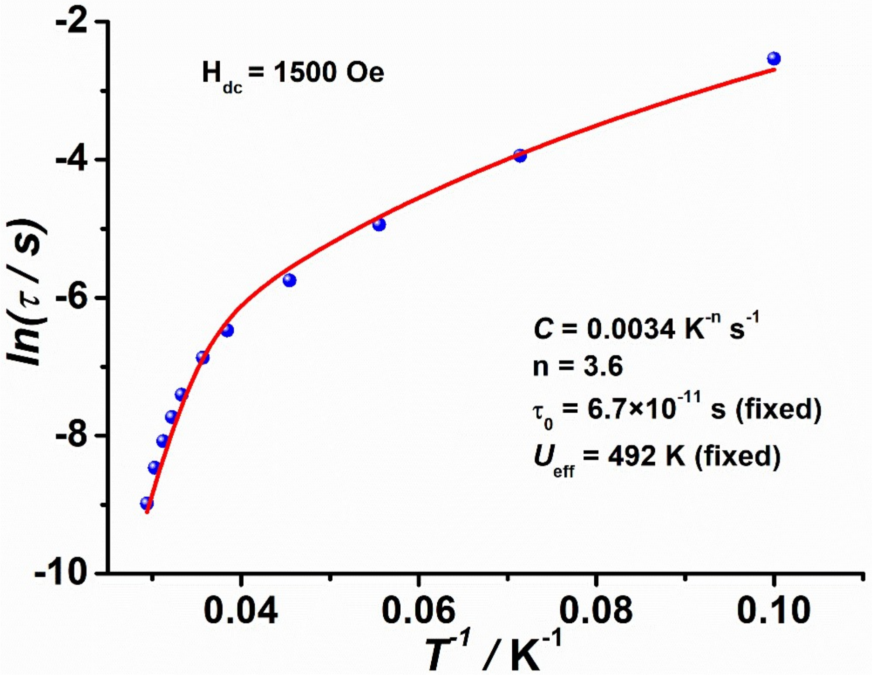

Figure S30. Plot of *ln(τ)* as functions of T^{-1} under 1500 Oe dc field for **3**. The green solid line is the best fit using the combination of Raman and Orbach processes based on the fixed values of U_{eff} = 492 K and $\tau_0 = 6.7 \times 10^{-11}$ s.

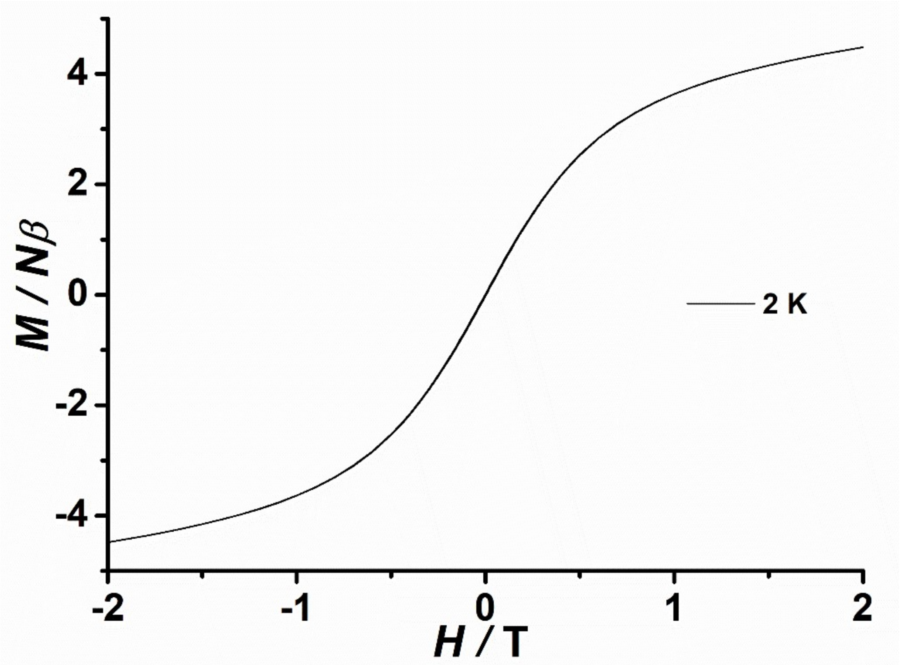

Figure S31. Powder magnetic hysteresis data for **1** at an average sweep rate of 0.02 T s^{-1}.

Theory Calculation

Table S8. Calculated energy levels (cm^{-1}), $\boldsymbol{g}$ (g_x, g_y, g_z) tensors and predominant m_J values of the lowest eight Kramers doublets (KDs) of complexes **1**–**3** using CASSCF/RASSI-SO with OpenMolcas.

KDs	1			2			3		
	E/cm^{-1}	$\boldsymbol{g}$	m_J	E/cm^{-1}	$\boldsymbol{g}$	m_J	E/cm^{-1}	$\boldsymbol{g}$	m_J
1	0.0	0.428<br>3.426<br>14.894	±15/2	0.0	0.001<br>0.002<br>19.857	±15/2	0.0	0.001<br>0.002<br>19.858	±15/2
2	18.5	0.131<br>2.841<br>9.728	±5/2	345.7	0.042<br>0.130<br>17.076	±13/2	317.5	0.279<br>0.491<br>16.877	±13/2
3	31.0	1.363<br>4.957<br>14.210	±3/2	459.6	2.694<br>4.931<br>11.865	±11/2	367.9	2.767<br>2.953<br>13.549	±7/2
4	98.6	0.935<br>1.339<br>14.626	±1/2	516.2	1.057<br>4.357<br>12.485	±5/2	417.8	0.177<br>3.140<br>14.849	±5/2
5	166.0	4.404<br>6.440<br>9.268	±13/2	566.3	4.094<br>4.779<br>12.145	±3/2	444.2	3.298<br>6.686<br>10.578	±3/2
6	226.7	2.429<br>2.767<br>15.227	±1/2	652.0	0.032<br>0.320<br>14.788	±1/2	498.6	0.473<br>1.175<br>16.116	±1/2
7	315.0	0.163<br>0.209<br>19.134	±13/2	752.9	0.075<br>0.910<br>18.474	±7/2	590.2	0.633<br>0.958<br>16.493	±9/2
8	442.9	0.042<br>0.132<br>19.579	±9/2	780.8	0.201<br>1.394<br>17.101	±5/2	629.9	0.415<br>1.623<br>17.325	±7/2

Table S9. Wave functions with definite projection of the total moment | m_J > for the lowest eight KDs of complexes **1**–**3**.

<table>
<tr><th></th><th>E/cm^{-1}</th><th>wave functions</th></tr>
<tr><td rowspan="8">1</td><td>0.0</td><td>63.1%|±15/2>+9.8%|±7/2>+8.9%|±3/2>+7.4%|±5/2></td></tr>
<tr><td>18.5</td><td>26.6%|±5/2>+24.4%|±7/2>+11.6%|±15/2>+9.6%|±3/2>+9.0%|±1/2>+8.4%|±9/2></td></tr>
<tr><td>31.0</td><td>21.6%|±3/2>+18.4%|±5/2>+16.0%|±1/2>+15.5%|±15/2>+12.0%|±9/2>+10.3%|±7/2></td></tr>
<tr><td>98.6</td><td>26.6%|±1/2>+23.6%|±9/2>+16.0%|±7/2>+15.8%|±11/2>+8.2%|±13/2></td></tr>
<tr><td>166.0</td><td>29.9%|±13/2>+23.8%|±3/2>+15.0%|±11/2>+11.6%|±5/2>+9.7%|±9/2></td></tr>
<tr><td>226.7</td><td>37.6%|±1/2>+16.6%|±13/2>+16.5%|±3/2>+15.9%|±11/2>+9.3%|±5/2></td></tr>
<tr><td>315.0</td><td>28.0%|±13/2>+24.5%|±11/2>+16.6%|±9/2>+13.1%|±7/2>+8.7%|±5/2></td></tr>
<tr><td>442.9</td><td>23.7%|±9/2>+22.0%|±7/2>+18.6%|±11/2>+15.7%|±5/2>+9.2%|±3/2></td></tr>
<tr><td rowspan="8">2</td><td>0.0</td><td>99.6%|±15/2></td></tr>
<tr><td>345.7</td><td>83.3%|±13/2>+8.9%|±9/2>+6.8%|±11/2></td></tr>
<tr><td>459.6</td><td>37.1%|±11/2>+33.9%|±7/2>+8.9%|±13/2>+7.7%|±9/2>+7.7%|±5/2></td></tr>
<tr><td>516.2</td><td>38.7%|±5/2>+28.2%|±9/2>+12.2%|±11/2>+8.1%|±3/2>+6.0%|±7/2></td></tr>
<tr><td>566.3</td><td>42.7%|±3/2>+25.7%|±1/2>+13.8%|±11/2>+6.8%|±5/2>+6.2%|±7/2></td></tr>
<tr><td>652.0</td><td>45.7%|±1/2>+16.7%|±9/2>+13.1%|±11/2>+10.9%|±3/2>+8.3%|±7/2></td></tr>
<tr><td>752.9</td><td>21.2%|±7/2>+20.4%|±3/2>+18.2%|±5/2>+17.0%|±1/2>+14.8%|±9/2></td></tr>
<tr><td>780.8</td><td>26.0%|±5/2>+24.0%|±7/2>+19.8%|±9/2>+14.6%|±3/2>+9.7%|±11/2></td></tr>
<tr><td rowspan="8">3</td><td>0.0</td><td>99.7%|±15/2></td></tr>
<tr><td>317.5</td><td>74.2%|±13/2>+15.2%|±9/2>+7.1%|±11/2></td></tr>
<tr><td>367.9</td><td>34.8%|±7/2>+17.1%|±5/2>+15.3%|±11/2>+13.9%|±13/2>+8.0%|±3/2></td></tr>
<tr><td>417.8</td><td>39.1%|±5/2>+14.8%|±11/2>+14.6%|±9/2>+14.3%|±7/2>+9.8%|±1/2></td></tr>
<tr><td>444.2</td><td>46.3%|±3/2>+23.2%|±1/2>+18.4%|±11/2></td></tr>
<tr><td>498.6</td><td>52.0%|±1/2>+17.3%|±3/2>+12.4%|±11/2>+10.5%|±9/2></td></tr>
<tr><td>590.2</td><td>28.2%|±9/2>+23.4%|±7/2>+17.3%|±11/2>+16.1%|±5/2>+8.1%|±3/2></td></tr>
<tr><td>629.9</td><td>23.1%|±7/2>+19.8%|±5/2>+19.6%|±9/2>+14.7%|±11/2>+13.5%|±3/2></td></tr>
</table>

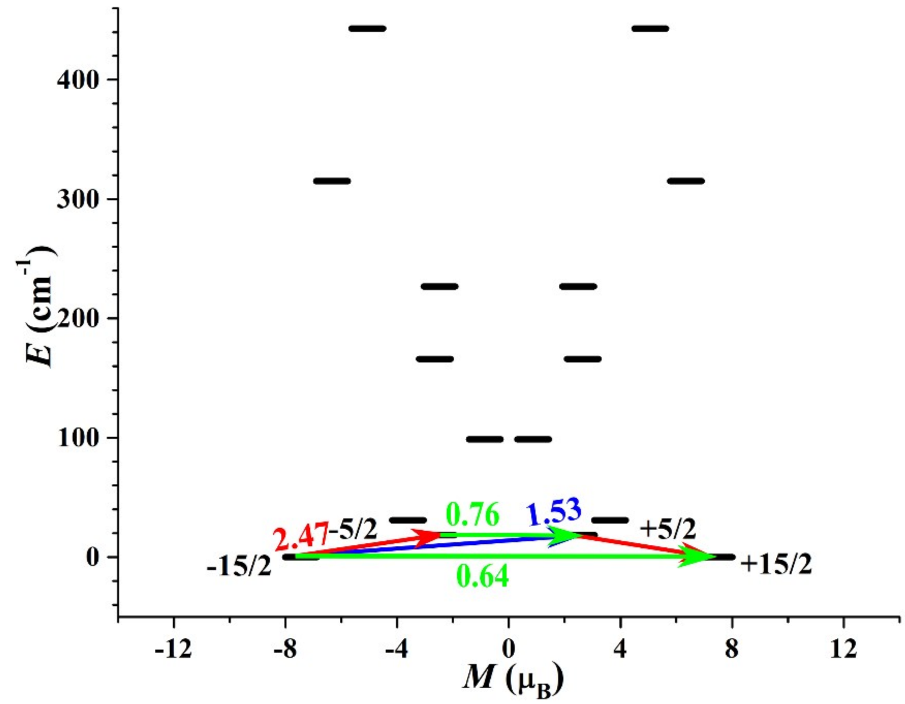

Figure S32. Magnetization-blocking barriers for complex **1**. The thick black lines represent KDs as function of magnetic moment along the magnetic axis. The green lines correspond to diagonal QTM, while the blue lines represent off-diagonal relaxation processes. The paths shown by the red arrows represents the most likely paths for magnetic relaxation in the corresponding compounds. The number associated with each arrow is the mean absolute value of the corresponding matrix element of the transition magnetic moment.

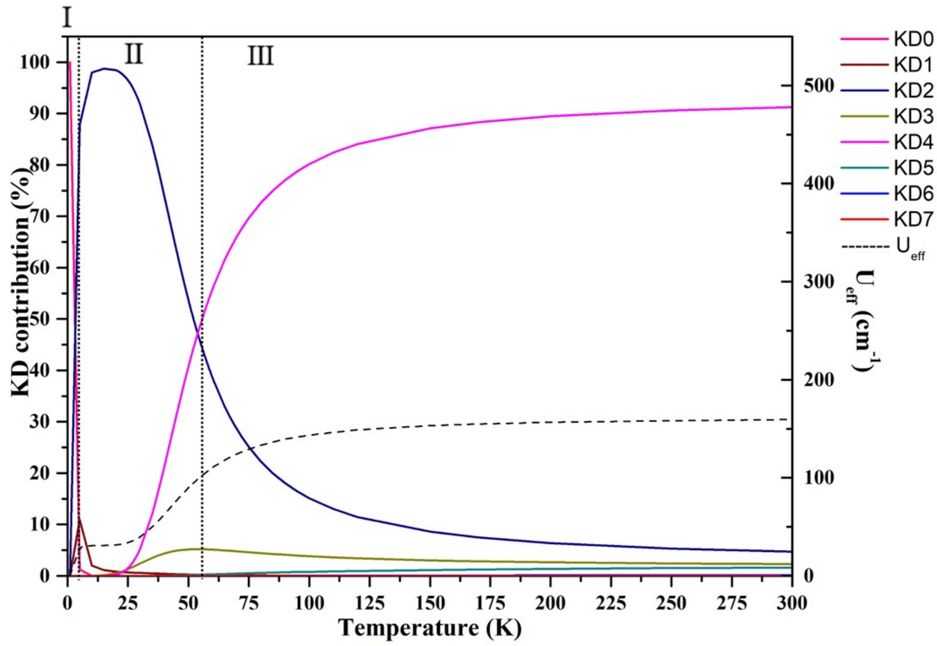

Figure S33. Predicted effective barrier and relaxation contributions from various KDs of complex **1**. Each U_{eff} is represented as a dashed black line, and its values is indicated on the right *y*-axis. The left *y*-axis represents the relative contribution of each KD to relaxation.

Table S10. Calculated crystal-field parameters *B* (*k*, *q*) and the corresponding weights for **1**–**3**.

**1**				**2**				**3**			
k	*q*	*B* (*k*, *q*)	Weight (%)	*k*	*q*	*B* (*k*, *q*)	Weight (%)	*k*	*q*	*B* (*k*, *q*)	Weight (%)
2	–2	–2.32	17.484	2	0	-0.33×10^{1}	22.09	2	0	-0.23×10^{1}	18.77
2	2	1.47	11.064	2	–2	-0.17×10^{1}	11.40	4	0	-0.86×10^{-2}	12.74
4	0	-0.51×10^{-2}	7.048	4	0	-0.87×10^{-2}	10.57	2	2	-0.16×10^{1}	12.61
4	–1	-0.44×10^{-2}	6.097	2	2	-0.14×10^{1}	9.62	2	–2	0.77	6.28
2	1	–0.70	5.302	2	1	–0.58	3.86	4	–2	0.32×10^{-2}	4.79
4	1	0.34×10^{-2}	4.707	4	–2	-0.30×10^{-2}	3.68	2	1	–0.53	4.30
6	–2	0.36×10^{-4}	4.645	6	2	0.31×10^{-4}	3.57	6	2	0.29×10^{-4}	3.99
4	–2	-0.31×10^{-2}	4.355	6	6	-0.31×10^{-4}	3.54	6	0	-0.26×10^{-4}	3.62
4	2	0.29×10^{-2}	4.075	6	–2	0.29×10^{-4}	3.27	4	2	-0.24×10^{-2}	3.56
6	–4	-0.27×10^{-4}	3.515	4	2	-0.27×10^{-2}	3.23	6	–2	-0.23×10^{-4}	3.16
6	0	-0.25×10^{-4}	3.251	6	1	0.27×10^{-4}	3.11	6	6	-0.20×10^{-4}	2.71
6	6	0.23×10^{-4}	3.037	4	–3	0.25×10^{-2}	2.99	4	–3	-0.17×10^{-2}	2.52
4	–4	-0.20×10^{-2}	2.807	6	–4	0.23×10^{-4}	2.63	6	–4	-0.18×10^{-4}	2.48
6	2	-0.20×10^{-4}	2.584	4	–4	0.21×10^{-2}	2.57	6	–6	-0.17×10^{-4}	2.31
2	0	–0.24	1.860	6	0	-0.21×10^{-4}	2.39	6	1	0.13×10^{-4}	1.82

Table S11. Calculated LoProp charges per atom in the ground KDs of complexes **2** and **3** using CASSCF with OpenMolcas.

		**2**	**3**
Easy axis	Dy	2.415	2.421
	O	–1.044	–0.995
	Average	–1.044	–0.995
Hard plane	N1	–0.395	–0.401
	N2	–0.409	–0.407
	N3	–0.319	–0.328
	N4	–0.397	–0.425
	N5	–0.385	–0.332
	N6	–0.415	–0.402
	N7	–0.325	–0.400
	Average	–0.377	–0.385
